# Supplementary material for: Treatment of diabetic kidney disease. A network meta-analysis
Source: PLoS One. 2023 Nov 2;18(11):e0293183. doi: 10.1371/journal.pone.0293183 (PMC10621862; doi:10.1371/journal.pone.0293183)
Supplement: S1 File — (PDF) [file pone.0293183.s001.pdf]

# S1 Baseline characteristics

| | Study Duration/ follow up time | Study-Drug | number of patients (study group) | Control-Drug | number of patients (control) | endpoints |
|---|---|---|---|---|---|---|
| ACEi+ARB vs. ACEi/ARB | | | | | | |
| Morgensen 2000 | 12 weeks | Candesartan + Lisinopril | 67 | G1: Candesartan; G2: Lisinopril | G1: 66; G2: 64 | albuminuria, hypotension |
| Tütüncü 2001 | 52 weeks | Enalapril + Losartan | 10 | G1: Enalapril; G2: Losartan | G1: 12; G2: 12 | albuminuria, hypotension |
| Atmaca 2006 | 52 weeks | Lisinopril + Losartan | 8 | G1: Lisinopril, G2: Losartan | G1: 9; G2:9 | albuminuria, hypotension |
| Ogawa 2007 | 48 weeks | G1: Temocapril + Candesartan; G2:Candesartan + Temocapril | G1: 37; G2: 35 | G1: Temocapril 4 mg; G2: Candesartan 8 mg | G1: 34; G2: 40 | albuminuria, hypotension |
| Titan 2011; NCT 00419835 | 17,5 weeks | Losartan + Enalapril | 28 | Enalapril | 28 | acute kidney injury, hyperkalemia |
| Fried 2013; NCT00555217 | median: 115 weeks | Losartan + Lisinopri | 724 | Losartan + Placebo | 724 | overall mortality, ESKD, acute kidney injury, hyperkalemia, hypotension |
| Imai 2013; NCT00141453 | 167 weeks | ACEi as before + Olmesartan | 205 | ACEi + Placebo | 209 | overall mortality, ESKD, acute kidney injury, hyperkalemia, hypotension |
| Fernandez Juarez 2013 | median follow up 139 weeks | Lisinopril + Irbesartan | 70 | G1:Lisinopril; G2: Irbesartan | G1: 35; G2: 28 | overall mortality, ESKD, hyperkalemia |
| Nakamura 2013 | 48 weeks | Losartan + Imidapril | 14 | Losartan | 14 | overall mortality, acute kidney injury |
| Saglimbene 2018 ; ACTRN12607000333415 | median follow up: 141 weeks | ACEi + ARB | 355 | G1: ACEi; G2: ARB | G1: 353; G2: 351 | overall mortality, ESKD, hyperkalemia, hypotension |
| Ruggenenti 2019, NCT00494715 | median 178 weeks | Benazepril + Valsartan | 33 | G1: Benazepril; G2: Valsartan | G1: 34; G2: 36 | overall mortality, ESKD, acute kidney injury, hyperkalemia, hypotension |

| | Study Duration/ follow up time | Study-Drug | number of patients (study group) | Control-Drug | number of patients (control) | endpoints |
|---|---|---|---|---|---|---|
| Direct Renin Inhibitors vs. ACEi/ARB | | | | | | |
| Persson 2010; NCT00097955 | median 26 weeks | Losartan + Aliskiren | 301 | Losartan + Placebo | 298 | overall mortality, acute kidney injury, albuminuria, hyperkalemia, hypotension |
| Bakris 2013, NCT00927394 | 8 weeks | Aliskiren + Valsartan | 80 | Valsartan | 65 | hyperkalemia, hypotension |
| Parving 2012, NCT00549757 | median 143 weeks | Aliskiren + ACE/ARB | 4274 | ACEi or ARB | 4287 | overall mortality, acute kidney injury, albuminuria, hyperkalemia, hypotension |
| Mineralocorticoidreceptor antagonists vs. ACEi/ARB | | | | | | |
| van den Meiracker 2006 | 52 weeks | Spironolacton + ACEi/ARB | 24 | ACEi/ARB+ Placebo | 29 | overall mortality, albuminuria, hyperkalemia |
| Epstein 2006 | 12 weeks | Eplerenone | 177 | Placebo | 91 | hyperkalemia, hypotension |
| Epstein 2002 | 24 weeks | | | | | hyperkalemia |
| Ziaee 2013; IRCT201105084849N2 | 12 weeks | Spironolacton + Enalapril | 29 | Enalapril | 31 | albuminuria |
| Kato 2015; UMIN 000008016 | 8 weeks | Spironolacton + own RASi (mostly used: telmisartan, olmesartan, valsartan)) | 26 | ACEi/ARB | 26 | albuminuria, hyperkalemia |
| Chen 2018 | 72 weeks | Spironolacton + Irbesartan | 55 | Irbesartan | 54 | albuminuria, hyperkalemia |
| El Mokadem 2020, NCT04143412 | 24 weeks | Ramipril + Eplerenone | 22 | Ramipril | 23 | albuminuria, hyperkalemia, hypotension |

| | **Study Duration/ follow up time** | **Study-Drug** | **number of patients (study group)** | **Control-Drug** | **number of patients (control)** | **endpoints** |
|---|---|---|---|---|---|---|
| SGLT 2 inhibitors vs. ACEi/ARB | | | | | | |
| Yale 2014, NCT01064414 | 52 weeks | Canagliflozin | 89 | Placebo | 90 | overall mortality, acute kidney injury, hyperkalemia, hypotension |
| Wanner 2020 , NCT01131676 | median duration of treatment 136 weeks | Empagliflozin | 509 | Placebo | 260 | overall mortality, acute kidney injury, hyperkalemia, hypotension |
| Fioretto 2018, NCT02413398 | 24 weeks | Dapagliflozin | 160 | Placebo | 161 | overall mortality, acute kidney injury, albuminuria, hypotension |
| Neuen 2019; CANVAS (NCT01032639);CANVAS -R (NCT01989754) | mean follow-up 188 weeks | Canagliflozin | 1728 | Placebo | 1298 | overall mortality, acute kidney injury, composite outcome, hyperkalemia, hypotension |
| Perkovic 2019; NCT 02065791 | median follow-up of 136 weeks | Canagliflozin | 2202 | Placebo | 2199 | overall mortality, ESKD, acute kidney injury, hyperkalemia, hypotension |
| Pollock 2019, NCT02547935 | 24 weeks | Dapagliflozin | 145 | Placebo | 148 | overall mortality, acute kidney injury, albuminuria, hypotension |

| | | | | | | |
|---|---|---|---|---|---|---|
| Bhatt 2020, NCT03315143 | 62 weeks (interquartile range 45 to 82) | Sotagliflozin | 5292 | placebo | 5292 | overall mortality, acute kidney injury, hypotension |

| | **Study Duration/ follow up time** | **Study-Drug** | **number of patients (study group)** | **Control-Drug** | **number of patients (control)** | **endpoints** |
|---|---|---|---|---|---|---|
| SGLT 2 inhibitors vs. ACEi/ARB | | | | | | |
| Mosenzon 2019, NCT01730534 | median follow-up of 218 weeks | Dapagliflozin | 2611 | placebo | 2588 | composite outcome |
| non-steroidal Mineralocorticoidreceptor antagonists vs. ACEi/ARB | | | | | | |
| Bakris 2015, NCT1874431 | 13 weeks | Finerenone | 117 | Placebo | 94 | overall mortality, hyperkalemia, hypotension |
| Katayama 2016; NCT01968668 | 13 weeks | Finerenone | alle 12 | Placebo | 12 | overall mortality |
| Ito 2019, NCT02345057 | 12 week | Esaxerenone | 70 | Placebo | 73 | acute kidney injury, albuminuria, hyperkalemia, hypotension |
| Ito 2020, JapicCTI-173695 | 52 weeks | Esaxerenone | 222 | Placebo | 227 | overall mortality, acute kidney injury, hyperkalemia |
| Bakris 2020; NCT02540993 | median follow-up of 136 weeks | Finerenone | 2833 | Placebo | 2841 | overall mortality, ESKD, acute kidney injury, composite outcome, hyperkalemia, hypotension |
| Pitt 2021, NCT02545049 | median follow-up of 177 weeks | Finerenone | 3686 | Placebo | 3666 | overall mortality, ESKD, acute kidney injury, composite outcome, hyperkalemia, hypotension |

| | Study Duration/ follow up time | Study-Drug | number of patients (study group) | Control-Drug | number of patients (control) | endpoints |
|---|---|---|---|---|---|---|
| multiarm trials | | | | | | |
| Mehdi 2009, NCT00381134 | 48 weeks | Losartan + Lisinopril | 26 | Spironolacton + Lisinopril | 27 | acute kidney injury, albuminuria, hyperkalemia, hypotension |
| Mehdi 2009, NCT00381134 | | | | Lisinopril | 27 | |
| ACEi+ARB vs. Mineralocorticoidreceptor antagonists | | | | | | |
| Esteghamati 2013, NCT01667614 | 78 weeks | Losartan + Enalapril | 62 | Losartan + Spironolacton | 74 | albuminuria, hyperkalemia |
| ACEi+ARB vs.Direct Renin Inhibitors | | | | | | |
| Imbalzano 2015 | 24 weeks | ACEi+Losartan or ARB+ramipril | 63 | ACEi or ARB + Aliskiren | 63 | albuminuria |

Baseline characteristics (Continuation)
Abbreviations: (SD Standard deviation) (25q/75q = 25th quartile, 75th quartile), IR = interquartile range, 95%CI = 95 confidence interval

| | Study group | Control group | Study group | Control group |
|---|---|---|---|---|
| | Age (in mean, if not stated differently) | | Sex (Male) (in mean, if not stated differently) | |
| ACEi+ARB vs. ACEi/ARB | | | | |
| Morgensen 2000 | 59,8 | G1: 59,7; G2: 59,9 | 55% | G1: 73%; G2: 67.2% |
| Tütüncü 2001 | 57,7 | G1: 51,4; G2: 58,1 | / | / |
| Atmaca 2006 | 55,1 | G2: 55,1; G2: 55,1 | 37,50% | G1: 44%, G2: 44% |
| Ogawa 2007 | G1: 61,8; G2: 62,5 | G1: 60,9; G2: 62,2 | G1:48,7%; G2: 48,6% | G1: 47%; G2: 47% |
| Titan 2011; NCT 00419835 | 58,1 | 58 | 71,40% | 54% |
| Fried 2013; NCT00555217 | 64,5 | 64,7 | 98,80% | 99,60% |
| Imai 2013; NCT00141453 | 59,1 | 59,3 | 70,40% | 67,50% |
| Fernandez Juarez 2013 | 63 | G1: 68,7; G2: 67,9 | 78% | G1: 70% G2: 75% |
| Nakamura 2013 | 61,7 | 61,4 | 71,40% | 64% |
| Saglimbene 2018 ; ACTRN12607000333415 | 64,1 | G1: 63; G2: 63,7 | 26,60% | G1: 38% G2: 27% |
| Ruggenenti 2019, NCT00494715 | 63,1 | G1: 66,3; G2: 63,9 | 81,80% | G1: 88.2% G2: 86.1% |
| Direct Renin Inhibitors vs. ACEi/ARB | | | | |
| Persson 2010; NCT00097955 | 59,8 | 61,8 | 68,40% | 74.2% |
| Bakris 2013, NCT00927394 | 55 | 55,2 | 59,40% | 56.8% |
| Parving 2012, | 64.6 (SD 9.6) | 64.4 (SD 9.9) | 67,4% | 68.7 % |
| Mineralocorticoidreceptor antagonists vs. ACEi/ARB | | | | |
| van den Meiracker 2006 | 55,2 | 55,2 | 66,70% | 58.6% |

| | | | | |
|---|---|---|---|---|
| Epstein 2006 | Median: 58 (25q/75q 52, 66) | Median: 60 (25q/75q 53, 66) | G1=66% G2= 65% | 55% |
| Epstein 2002 | / | / | / | / |
| Ziaee 2013; IRCT201105084849N2 | 53,1 | 53,03 | 64,50% | 66.5% |
| Kato 2015; UMIN 000008016 | 61 | 59,4 | 69,20% | 73.8% |
| Chen 2018 | G1: 67; G2: 67 | G1: 68; G2: 67 | G1: 48,1%; G2: 53,1% | G1: 52.8%; G2: 51.9% |
| El Mokadem 2020, NCT04143412 | 48,48 | 50,4 | 60% | 52% |
| SGLT 2 inhibitors vs. ACEi/ARB | | | | |
| Yale 2014, NCT01064414 | 67.9 (SD 8.2) | 68.2 (SD 8.4) | 53.9% | 63.3% |
| Wanner 2020 , NCT01131676 | 63.6 (SD 8.4) | / | 76.1% | / |
| Fioretto 2018, NCT02413398 | 66.0 | 66.2 | 56.9% | 56.5% |
| Neuen 2019; CANVAS (NCT01032639);CANVAS-R (NCT01989754) | G1: 63.8 (SD 8.3); G2: 63.4 (SD 8.3) | P1: 64.2 (SD 8.3); P2: 64 (SD 8.2) | G1 70.8%; G2 70% | P1 67.6%; P2 70.1% |
| Perkovic 2019; NCT 02065791 | 62.9 (SD 9.2) | 63.2 (SD 9.2) | 65,40% | 66,70% |
| Pollock 2019, NCT02547935 | 64.7 (SD 8.6) | 64.7 (SD 8.5) | 70% | 71% |
| Bhatt 2020, NCT03315143 | Meadian 69 (IR 63-74) | Meadian 69 (IR 63–74) | 55.7% | 54.5% |
| Mosenzon 2019, NCT01730534 | / | / | / | / |
| non-steroidal Mineralocorticoidreceptor antagonists vs. ACEi/ARB | | | | |
| Bakris 2015, NCT1874431 | 64.70 (SD 9.26) | 63.26 (SD 8.68) | 74.8% | 73.4% |
| Katayama 2016; NCT01968668 | 64.00 (SD 8.26) | 66.75 (SD 9.02) | 66.7% | 83.3% |
| Ito 2019, NCT02345057 | 64 (SD 11) | 66 (SD 10) | 81% | 78% |

| | | | | |
|---|---|---|---|---|
| Ito 2020, JapicCTI-173695 | 66 (SD 10) | 66 (SD 9) | 74% | 79% |
| Bakris 2020; NCT02540993 | 65.4 (SD 8.9) | 65.7 (SD 9.2) | 68.9% | 71.5% |
| Pitt 2021, NCT02545049 | 64.1 (SD 9.7) | 64.1 (SD 10.0) | 68.6% | 70.3% |
| multiarm trials | | | | |
| Mehdi 2009, NCT00381134 | 52,3 | C1: 52,7 | 50% | C1: 48.10% |
| Mehdi 2009, NCT00381134 | | C2: 49,3 | | C2: 44% |
| ACEi+ARB vs. Mineralocorticoidreceptor antagonists | | | | |
| Esteghamati 2013, NCT01667614 | 58,33 | 57,8 | 64.5% | 68.90% |
| ACEi+ARB vs.Direct Renin Inhibitors | | | | |
| Imbalzano 2015 | 66,4 | 67,2 | 50.8% | 52.4% |

Baseline characteristics (Continuation)
Abbreviations: (SD Standard deviation) (25q/75q = 25th quartile, 75th quartile), IR = interquartile range, 95%CI = 95 confidence interval

| | Study group | Control group | Study group | Control group |
|---|---|---|---|---|
| | eGFR(base) (ml/min) (in mean, if not stated differently) | | UACR(base) (mg/gCrea ) (in mean, if not stated differently) | |
| ACEi+ARB vs. ACEi/ARB | | | | |
| Morgensen 2000 | 98.4 (SD 32.9) | G1: 103.5 (SD 38.4); G2: 96.8 (SD 28.9) | 49,504 | G1: 63,65; G2: 52,16 |
| Tütüncü 2001 | / | / | 102,03 mg/d (SD 32,77) | G1: 85,02 mg/d (SD 31,25); G2: 101,66 (SD 41,19) |
| Atmaca 2006 | / | / | / | / |
| Ogawa 2007 | / | / | geometric mean G1: 235 (range 123–282); G2: 261 (range 126–267) | geometric mean G1: 245 (range 108–286); G2: 238 (range 134–285) |
| Titan 2011; NCT 00419835 | 52,6 | 53,2 | / | / |
| Fried 2013; NCT00555217 | 53.6 (SD 15.5) | 53.7 (SD 16.2) (falls benötigt gibt es hier auch Aufteilung nach CKD Stadien) | median 842 (IR 495 –1698) | median 862 (IR 488–1789) |
| Imai 2013; NCT00141453 | 37.25 (SD 9.64) | 37.07 (SD 10.03) | median 1700 (IR 770 - 2990) | median 1690 (IR 870 - 3120) |
| Fernandez Juarez 2013 | 50 (SD 25) | G1: 48 (SD 14); G2: 46 (SD 16) | / | / |
| Nakamura 2013 | CreaClearance: 90,1 (SD 36,1) | CreaClearance: 85,4 (SD 16,1) | 280 (SD 202) | 234 (SD 197) |

| | | | | |
|---|---|---|---|---|
| Saglimbene 2018 ; ACTRN12607000333415 | 67.0 (SD 27.4) | G1: 112 (SD 34.3); G2: 129 (SD 39.7) | median 120 (IR 56–282) | G1: median 102 (IR 53–280); G2: median 103 (IR 52–294) |
| Ruggenenti 2019, NCT00494715 | median 39.7 (IR 31.9–49.0) (mGFR) | G1: median 39.9 (IR 29.7–47.5); G2: median 42.0 (IR 34.4–69.1) mGFR | / | / |
| Direct Renin Inhibitors vs. ACEi/ARB | | | | |
| Persson 2010; NCT00097955 | 68,5 (SD 25,7) | 66,8 (SD 24,5) | 513 | 553 |
| Bakris 2013, NCT00927394 | G1: 89.6 (SD 20.4); G2: 91.4 (SD 20.8) | G1: 94.1 (SD 21.6); G2: 88.9 (SD 18.9) | / | / |
| Parving 2012, | 57.0 (SD 21.9) | 57.0 (SD 23.0) | geometric mean 206 (IR 57- 866) | geometric mean 208 (IR 58 - 912) |
| Mineralocorticoidreceptor antagonists vs. ACEi/ARB | | | | |
| van den Meiracker 2006 | geometric mean: 87 (IR 67–109) | geometric mean: 64 (IR 47–87) | geometric mean 571,06 (IR 292,6–952,07) | geometric mean 898,14 (IR 386,31-2519,4) |
| Epstein 2006 | G1: 73 (SD 62.1, 83.6) G2: 75 (SD 62.8, 85.9) | 74 (SD 60.5, 82.2) | median G1: 422 (25q/75q 153.8, 856.0), G2: 240 (25q/75q 90.7, 577.8) | median 280 (25q/75q 105.1, 762.2) |
| Epstein 2002 | / | / | / | / |

| | | | | |
|---|---|---|---|---|
| Ziaee 2013; IRCT201105084849 N2 | 79.84 (SD 18.05) | 82.55 (SD 19.18) | 126.3 (SD 69.36) | 119 (SD 66.86) |
| Kato 2015; UMIN 000008016 | / | / | 702.1 (SD 728) | 511 (SD 450) |
| Chen 2018 | G1: 79.3 (SD 13.9); G2: 80.0 (SD 13.5) | G1: 79.1 (SD 12.5), G2: 78.7 (SD 12.7) | / | / |
| El Mokadem 2020, NCT04143412 | 72.84 (SD 11.93) | 71.8 (SD 8.46) | 165.40 (SD 62.94) | 159.96 (SD 66.22) |
| SGLT 2 inhibitors vs. ACEi/ARB | | | | |
| Yale 2014, NCT01064414 | 38.5 (SD 6.9) | 40.1 (SD 6.8) | 228.1 (SD 603.9) | 255.1 (SD 468.8) |
| Wanner 2020 , NCT01131676 | G1: 64.9 (SD 21.0); G2: 47.7 (SD 7.5) | / | geometric mean 877 (25q/75q 441, 1461) | / |
| Fioretto 2018, NCT02413398 | 53.3 (SD 8.7) | 53.6 (SD 10.6) | median 23.5 (IR 2.7–5852.0) | median 29.0 (IR 3.8-8474.0) |
| Neuen 2019; CANVAS (NCT01032639);CANVAS-R (NCT01989754) | G1 74.8 (SD 20.9); G2 65.9 (SD 22.2) | P1:73.9 (SD 21.9); P2:66.9 (SD 22.5) | median G1: 67.1 (IR 42.6- 127.2) ; G2 691.9 (IR 433.2- 1255.4) | median P1: 69.4 (IR 44.6–120.5); P2: 763.2 (IR 451.5–1394.1) |
| Perkovic 2019; NCT 02065791 | 56.3 (SD18.2) | 56.0 (SD 18.3) | median 923.0 (IR 459–1794) | median 931 (IR 473–1868) |
| Pollock 2019, NCT02547935 | 50.2 (SD 13.0) | 47.7 (SD 13.5) | median 270.0 (IR 69–751) | median 257.5 (IR 80–949) |
| Bhatt 2020, NCT03315143 | Meadian 44.4 (IR 37.0–51.3) | Median 44.7 (IR 37.0–51.5) | median 74 (IR 18–486) | median 75 (IR 17–477) |
| Mosenzon 2019, NCT01730534 | / | / | / | / |

| | | | | | |
|---|---|---|---|---|---|
| non-steroidal Mineralocorticoidreceptor antagonists vs. ACEi/ARB | | | | | |
| Bakris 2015, NCT1874431 | 66.0 (SD 22.2) | 72.2 (SD 20.4) | | median 202.7 (IR 4.4-2298) | median 182. 9 (IR 15.0-3056) |
| Katayama 2016; NCT01968668 | 61.48 (SD 11.01) | 60.88(SD 16.53) | | median 228.67 (IR 51.3–1917.4) | median 287.74 (IR 28.9–1338.1) |
| Ito 2019, NCT02345057 | 68 (SD 19) | 69 (SD 19) | | geometric mean (95% CI) 112((100 to 126) | geometric mean (95% CI) 110 (98 to 123) |
| Ito 2020, JapicCTI-173695 | 69(SD 18) | 69 (SD 18) | | Median (min, max) 113 (46, 286) | Median (max, min) 110 (47, 278) |
| Bakris 2020; NCT02540993 | 44.4 (SD 12.5) | 44.3 (SD 12.6) | | Median (IQR) 833 (441–1628) | 867 (453–1645)Median (IQR) |
| Pitt 2021, NCT02545049 | 67.6 (SD 21.7) | 68.0 (SD 21.7) | | 302 (105–749) Median IQR | 315 (111–731) Median IQR |
| multiarm trials | | | | | |
| Mehdi 2009, NCT00381134 | / | / | | geometric mean: 897 (95% CI: 611–1316) | C1: geometric mean 1094 (95% CI 758–1579) |
| Mehdi 2009, NCT00381134 | | / | | | C2: geometric mean 917 (95% CI633–1329) |
| ACEi+ARB vs. Mineralocorticoidreceptor antagonists | | | | | |

| | | | | |
|---|---|---|---|---|
| Esteghamati 2013, NCT01667614 | 69.13 (SD 19.69) | 73.75 (SD 16.78) | median 95.0 (IR 50.0, 330.0) | median 112.0 (IR 63.5, 317.3) |
| ACEi+ARB vs.Direct Renin Inhibitors | | | | |
| Imbalzano 2015 | Crea-Clearance: 73.0 (SD 19.8) | Crea-Clearance: 75.30 (SD 19.8) | 103.4 (SD 50.0) | 103.4 (SD 56.8) |
