# Supplementary material for: Treatment of diabetic kidney disease. A network meta-analysis
Source: PLoS One. 2023 Nov 2;18(11):e0293183. doi: 10.1371/journal.pone.0293183 (PMC10621862; doi:10.1371/journal.pone.0293183)
Supplement: S2 File — (PDF) [file pone.0293183.s002.pdf]

S2 Citation list

1. Mogensen CE, Neldam S, Tikkanen I, Oren S, Viskoper R, Watts RW, et al. Randomised controlled trial of dual blockade of renin-angiotensin system in patients with hypertension, microalbuminuria, and non-insulin dependent diabetes: the candesartan and lisinopril microalbuminuria (CALM) study. BMJ. 2000 Dec;321(7274):1440–4.
2. Tütüncü NB GA, Gedik O. Efficacy of ACE inhibitors and ATII receptor blockers in patients with microalbuminuria: a prospective study. Acta Diabetol. 38(4).
3. Atmaca A, Gedik O, Atmaca A, Gedik O. Effects of angiotensin-converting enzyme inhibitors, angiotensin II receptor blockers, and their combination on microalbuminuria in normotensive patients with type 2 diabetes. Adv Ther. 2006;23(4):615–22.
4. Ogawa S, Takeuchi K, Mori T, Nako K, Tsubono Y, Ito S. Effects of monotherapy of temocapril or candesartan with dose increments or combination therapy with both drugs on the suppression of diabetic nephropathy. Hypertens Res. 2007 Apr;30(4):325–34.
5. Titan SM, M Vieira JJ, Dominguez W V, Barros RT, Zatz R, Titan SM MVJDWVBRT, et al. ACEI and ARB combination therapy in patients with macroalbuminuric diabetic nephropathy and low socioeconomic level: a double-blind randomized clinical trial. Clin Nephrol. 2011 Oct;76(4):273–83.
6. Fried LF, Emanuele N, Zhang JH, Brophy M, Conner TA, Duckworth W, et al. Combined angiotensin inhibition for the treatment of diabetic nephropathy. N Engl J Med. 2013 Nov;369(20):1892–903.
7. Imai E, Haneda M, Yamasaki T, Kobayashi F, Harada A, Ito S, et al. Effects of dual blockade of the renin-angiotensin system on renal and cardiovascular outcomes in type 2 diabetes with overt nephropathy and hypertension in the ORIENT: a post-hoc analysis (ORIENT-Hypertension). Hypertens Res. 2013 Dec;36(12):1051–9.
8. Fernandez Juarez G, Luño J, Barrio V, de Vinuesa SG, Praga M, Goicoechea M, et al. Effect of dual blockade of the renin-angiotensin system on the progression of type 2 diabetic nephropathy: a randomized trial. Am J kidney Dis Off J Natl Kidney Found. 2013 Feb;61(2):211–8.
9. Nakamura A, Shikata K, Nakatou T, Kitamura T, Kajitani N, Ogawa D, et al. Combination therapy with an angiotensin-converting-enzyme inhibitor and an angiotensin II receptor antagonist ameliorates microinflammation and oxidative stress in patients with diabetic nephropathy. J Diabetes Investig. 2013 Mar;4(2):195-201.
10. Saglimbene V, Palmer SC, Ruospo M, Natale P, Maione A, Nicolucci A, et al. The long-term impact of renin-angiotensin system (RAS) inhibition on cardiorenal outcomes (LIRICO): A randomized, controlled trial. J Am Soc Nephrol. 2018;29(12):2890–9.
11. Ruggenenti P, Trillini M, P Barlovic D, Cortinovis M, Pisani A, Parvanova A, et al. Effects of valsartan, benazepril and their combination in overt nephropathy of type 2 diabetes: A prospective, randomized, controlled trial. Diabetes Obes Metab. 2019 May;21(5):1177–90.
12. Persson F, Lewis JB, Lewis EJ, Rossing P, Hollenberg NK, Parving H-H. Impact of baseline renal function on the efficacy and safety of aliskiren added to losartan in patients with type 2 diabetes and nephropathy. Diabetes Care. 2010 Nov;33(11):2304–9.
13. Bakris GL, Oparil S, Purkayastha D, Yadao AM, Alessi T, Sowers JR, et al. Randomized study of antihypertensive efficacy and safety of combination aliskiren/valsartan vs valsartan monotherapy in hypertensive participants with type 2 diabetes mellitus. J Clin Hypertens (Greenwich). 2013 Feb;15(2):92–100.
14. Parving H-H, Brenner BM, McMurray JJV, de Zeeuw D, Haffner SM, Solomon SD, et al. Cardiorenal End Points in a Trial of Aliskiren for Type 2 Diabetes. N Engl J Med. 2012;367(23):2204–13.
15. van den Meiracker AH, Baggen RGAG, Pauli S, Lindemans A, Vulto AG, Poldermans D, et al. Spironolactone in type 2 diabetic nephropathy: Effects on proteinuria, blood pressure and renal function. J Hypertens. 2006 Nov;24(11):2285–92.
16. Epstein M, Williams GH, Weinberger M, Lewin A, Krause S, Mukherjee R, et al. Selective aldosterone blockade with eplerenone reduces albuminuria in patients with type 2 diabetes. Clin J Am Soc Nephrol. 2006;1(5):940–51.

17. or-55 Comparison of Eplerenone and Losartan in Patients With Low-Renin Hypertension or-54 Antiproteinuric Efficacy of Eplerenone , Enalapril , and Eplerenone / Enalapril Combination Therapy in Diabetic. 2002;15(4):2002.
18. Ziaee A, Abbas Vaezi A, Oveisi S, Javadi A, Hashemipour S, Kazemifar AM, et al. Effects of additive therapy with spironolactone on albuminuria in diabetes mellitus: a pilot randomized clinical trial. Casp J Intern Med. 2013;4(2):648-653.
19. Kato S, Matsuo S, Maruyama S, Makino H, Wada J, et al. Anti-albuminuric effects of spironolactone in patients with type 2 diabetic nephropathy: a multicenter, randomized clinical trial. Clin Exp Nephrol. 2015 Dec;19(6):1098-1106.
20. Chen Y, Liu P, Chen X, Li Y, Zhang F, Wang Y, et al. Effects of Different Doses of Irbesartan Combined With Spironolactone on Urinary Albumin Excretion Rate in Elderly Patients With Early Type 2 Diabetic Nephropathy. Am J Med Sci [Internet]. 2018 May;355(5):418-424. Available from: https://doi.org/10.1016/j.amjms.2018.01.017
21. El Mokadem M, Abd El Hady Y, Aziz A. A Prospective Single-Blind Randomized Trial of Ramipril, Eplerenone and Their Combination in Type 2 Diabetic Nephropathy. Cardiorenal Med. 2020;10(6):392–401.
22. Yale J-FF, Bakris G, Cariou B, Nieto J, David-Neto E, Yue D, et al. Efficacy and safety of canagliflozin over 52 weeks in patients with type 2 diabetes mellitus and chronic kidney disease. Diabetes Obes Metab. 2014 Oct;16(10):1016-1027.
23. Heerspink HJLL, Wheeler DC, Stefánsson B V., Correa-Rotter R, Chertow GM, et al. Dapagliflozin in Patients with Chronic Kidney Disease. N Engl J Med. 2020 Oct;383(15):1436-1446.
24. Fioretto P, Del Prato S, Buse JB, Goldenberg R, Giorgino F, Reyner D, et al. Efficacy and safety of dapagliflozin in patients with type 2 diabetes and moderate renal impairment (chronic kidney disease stage 3A): the DERIVE Study. Diabetes Obes Metab. 2018 Nov;20(11):2532-2540.
25. Neuen BL, Perkovic V, Ohkuma T, Neal B, Matthews DR, et al. Effect of Canagliflozin on Renal and Cardiovascular Outcomes across Different Levels of Albuminuria: data from the CANVAS Program. J Am Soc Nephrol. 2019 Nov;30(11):2229-2242.
26. Perkovic V, Jardine MJ, Neal B, Bompoint S, Heerspink HJLL, Charytan DM, et al. Canagliflozin and Renal Outcomes in Type 2 Diabetes and Nephropathy. N Engl J Med. 2019 Jun;380(24):2295-2306.
27. Pollock C, Stefánsson B, Reyner D, Rossing P, Sjöström CD, Wheeler DC, et al. Albuminuria-lowering effect of dapagliflozin alone and in combination with saxagliptin and effect of dapagliflozin and saxagliptin on glycaemic control in patients with type 2 diabetes and chronic kidney disease (DELIGHT): a randomised, double-blind, plac. lancet Diabetes Endocrinol. 2019 Jun;7(6):429-441.
28. Bhatt DL, Steg PG, Szarek M, Pitt B, Cannon CP, et al. Sotagliflozin in Patients with Diabetes and Chronic Kidney Disease. N Engl J Med. 2021 Jan;384(2):129-139.
29. Mosenzon O, Wiviott SD, Cahn A, Rozenberg A, Yanuv I, Goodrich EL, et al. Effects of dapagliflozin on development and progression of kidney disease in patients with type 2 diabetes: an analysis from the DECLARE-TIMI 58 randomised trial. lancet Diabetes Endocrinol. 2019 Aug;7(8):606-617.
30. Bakris GL, Agarwal R, Chan JC, Cooper ME, Gansevoort RT, Haller H, et al. Effect of finerenone on albuminuria in patients with diabetic nephropathy a randomized clinical trial. JAMA - J Am Med Assoc. 2015;314(9):884–94.
31. Katayama S, Yamasaki Y, Yamada D, Nakayama M, Yamada T, et al. A randomized controlled study of finerenone versus placebo in Japanese patients with type 2 diabetes mellitus and diabetic nephropathy. J Diabetes Complications [Internet]. 2017 Apr;31(4):758–65. Available from: http://dx.doi.org/10.1016/j.jdiacomp.2016.11.021
32. Ito S, Sawanobori T, Shikata K, Nangaku M, Okuda Y, et al. Efficacy and Safety of Esaxerenone (CS-3150) for the Treatment of Type 2 Diabetes with Microalbuminuria: a Randomized, Double-Blind, Placebo-Controlled, Phase II Trial. Clin J Am Soc Nephrol. 2019;14(8):1161-1172.
33. Ito S, Sawanobori T, Kashihara N, Shikata K, Nangaku M, et al. Esaxerenone (CS-3150) in patients with type 2 diabetes and microalbuminuria (ESAX-DN): phase 3 randomized controlled clinical trial. Clin J Am Soc Nephrol. 2020 Dec;15(12):1715-1727.

34. Bakris GL, Agarwal R, Anker SD, Pitt B, Ruilope LM, Rossing P, et al. Effect of Finerenone on Chronic Kidney Disease Outcomes in Type 2 Diabetes. N Engl J Med. 2020;383(23):2219–29.
35. Pitt B, Filippatos G, Agarwal R, Anker SD, Bakris GL, Rossing P, et al. Cardiovascular Events with Finerenone in Kidney Disease and Type 2 Diabetes. N Engl J Med [Internet]. 2021 Aug 28;385(24):2252–63. Available from: https://doi.org/10.1056/NEJMoa2110956
36. Mehdi UF, Toto RD, Adams-Huet B, Raskin P, Vega GL, et al. Addition of angiotensin receptor blockade or mineralocorticoid antagonism to maximal angiotensin-converting enzyme inhibition in diabetic nephropathy. J Am Soc Nephrol. 2009 Dec;20(12):2641–50.
37. Imbalzano E, Scarpelli M, Mandraffino G, Creazzo M, Lizio G, Trapani G, et al. Combination therapy with aliskiren versus ramipril or losartan added to conventional therapy in patients with type 2 diabetes mellitus, uncontrolled hypertension and microalbuminuria. J Renin Angiotensin Aldosterone Syst. 2015 Dec;16(4):956–64.
