# Supplementary material for: Treatment of diabetic kidney disease. A network meta-analysis
Source: PLoS One. 2023 Nov 2;18(11):e0293183. doi: 10.1371/journal.pone.0293183 (PMC10621862; doi:10.1371/journal.pone.0293183)
Supplement: S4 File — (PDF) [file pone.0293183.s004.pdf]

<u>S4 Search strategy</u>

Full search terms for PUBMED

non-steroidal MRA: Finerenone & Esaxerenone
Finerenone AND kidney
(61 results)
esaxerenone AND Kidney
(13 results)

DRIs (direct renin inhibitors): Aliskiren
(Aliskiren OR DRI) AND (diabetic nephropathy OR CKD OR diabetes) AND (RCT OR randomized)
(121 results)

Aldosteron antagonists: Spironolactone / Eplerenone
(spironolactone OR Eplerenone OR Aldosterone antagonist) AND (diabetic nephropathy OR Chronic kidney disease OR CKD) AND (RCT OR randomized) 218 results

ACE + ARB combination
(ACE inhibitor OR Benazapril OR Captopril OR Cilazapril OR Enalapril OR Fosinopril OR Imidapril OR Lisinopril OR Moexipril OR Perindopril OR Quinapril OR Ramipril OR Trandolapril) AND (ARB OR Candesartan OR Eprosartan OR Irbesartan OR Losartan OR Olmesartan OR Telmisartan OR Valsartan OR Angiotensin receptor blockers OR angiotensin II receptor antagonists OR Azilsartan OR Eprosartan)
AND (diabetic nephropathy OR chronic kidney disease OR CKD) AND (RCT OR randomized)
(660 results)

SGLT- 2 inhibitors
(SGLT2-inhibitor OR Canagliflozin OR Dapagliflozin OR Empagliflozin OR Ertugliflozin OR Ipragliflozin OR Remogliflozin OR Sergliflozin OR Sotagliflozin OR Tofogliflozin) AND (diabetic nephropathy OR (diabetes AND kidney) OR chronic kidney disease OR CKD) AND (RCT OR randomized)
(423 results)
