# Supplementary material for: Treatment of diabetic kidney disease. A network meta-analysis
Source: PLoS One. 2023 Nov 2;18(11):e0293183. doi: 10.1371/journal.pone.0293183 (PMC10621862; doi:10.1371/journal.pone.0293183)
Supplement: S5 File — (PDF) [file pone.0293183.s005.pdf]

S5 Tests of heterogeneity (within designs) and inconsistency (between designs) with Cochran's *Q* statistics:

Overall mortality

	Q	d.f.	p-value
Total	16.47	13	0.2246
Within designs	16.47	13	0.2246
Between designs	0.00	0	--

End stage kidney disease

	Q	d.f.	p-value
Total	3.48	5	0.6258
Within designs	3.48	5	0.6258
Between designs	0.00	0	--

Renal composite outcome

	Q	d.f.	p-value
Total	0.64	2	0.7254
Within designs	0.64	2	0.7254
Between designs	0.00	0	--

Albuminuria

	Q	d.f.	p-value
Total	49.89	13	< 0.0001
Within designs	42.95	9	< 0.0001
Between designs	6.94	4	0.1390

Acute kidney injury

	Q	d.f.	p-value
Total	8.53	13	0.8074
Within designs	8.53	12	0.7424
Between designs	0.00	1	0.9869

Hyperkalemia

	Q	d.f.	p-value
Total	29.23	20	0.0833
Within designs	25.81	17	0.0779
Between designs	3.42	3	0.3316

Hypotension

	Q	d.f.	p-value
Total	24.05	16	0.0885
Within designs	23.40	15	0.0760
Between designs	0.64	1	0.4223
