# Supplementary material for: Treatment of diabetic kidney disease. A network meta-analysis
Source: PLoS One. 2023 Nov 2;18(11):e0293183. doi: 10.1371/journal.pone.0293183 (PMC10621862; doi:10.1371/journal.pone.0293183)

S6 Distribution of direct and indirect evidence

Overall Mortality

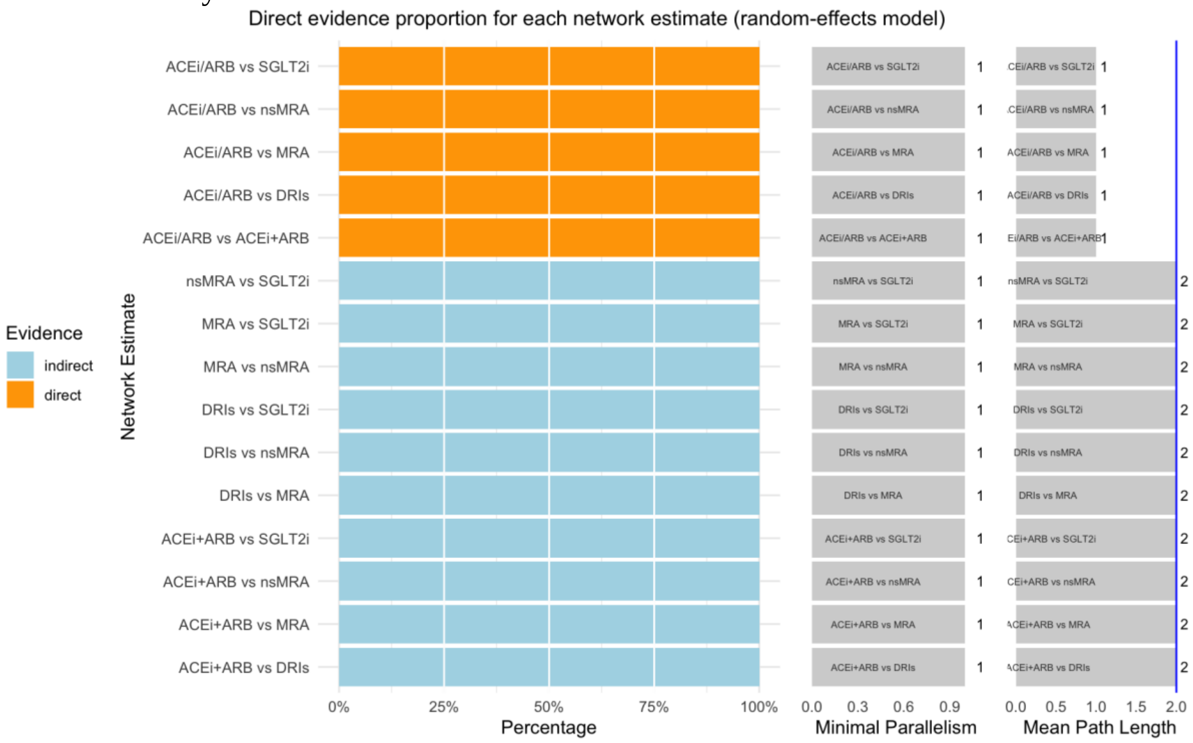

End stage kidney disease

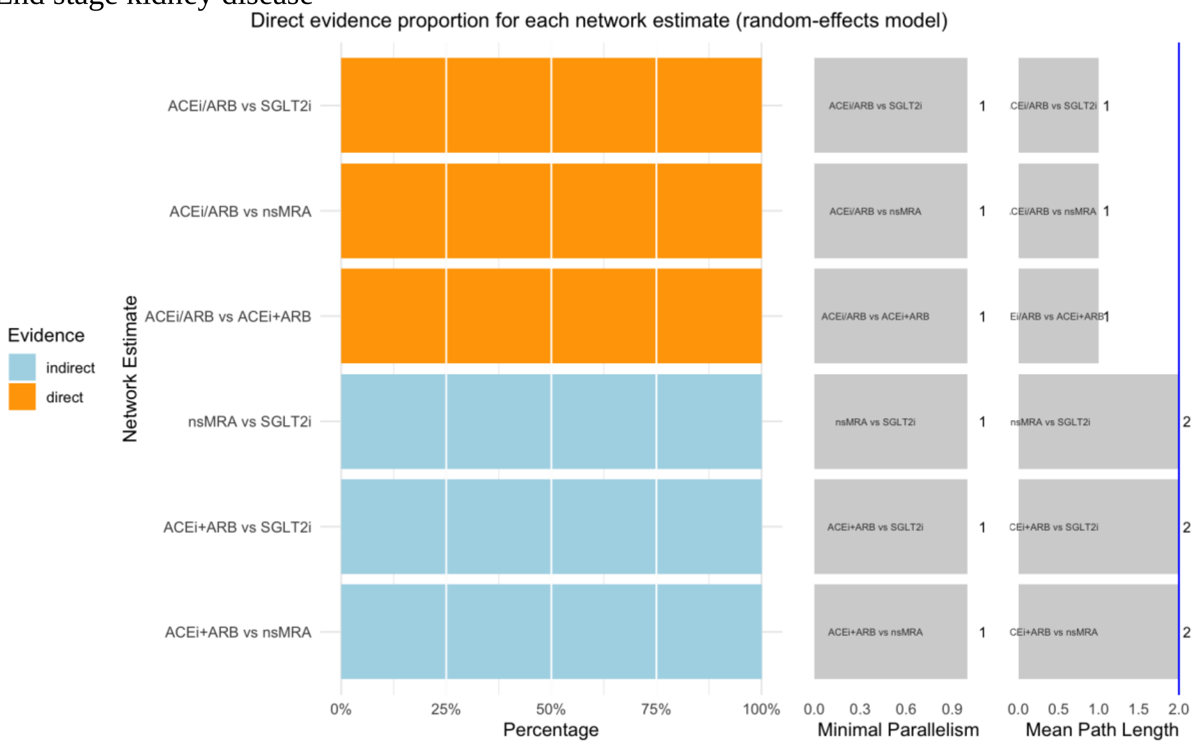

Albuminuria

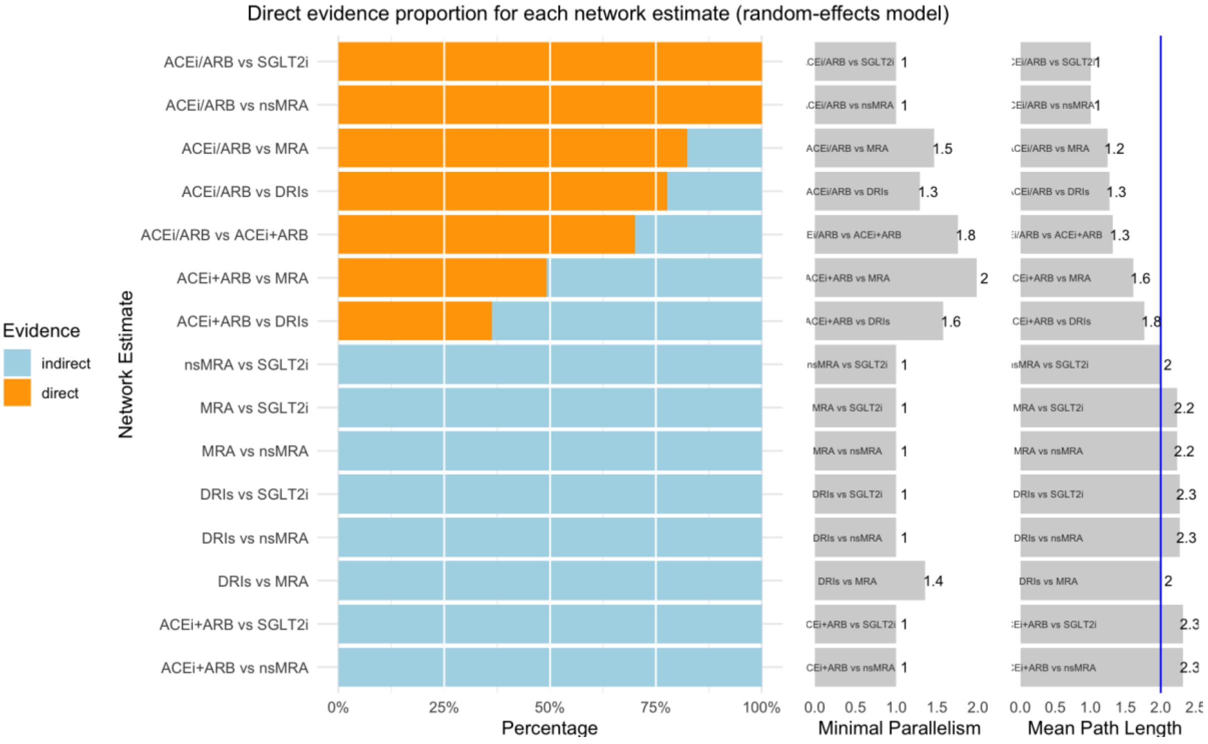

Renal composite outcome

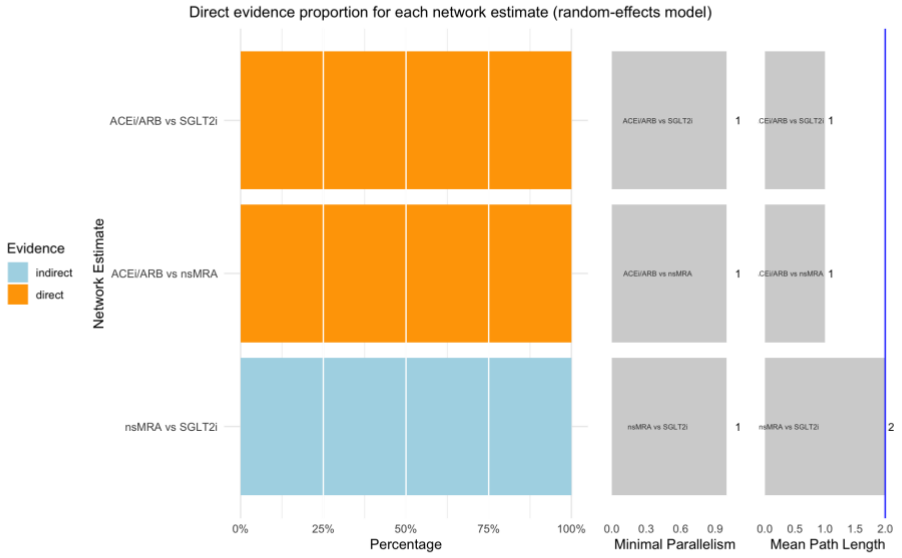

Acute kidney injury

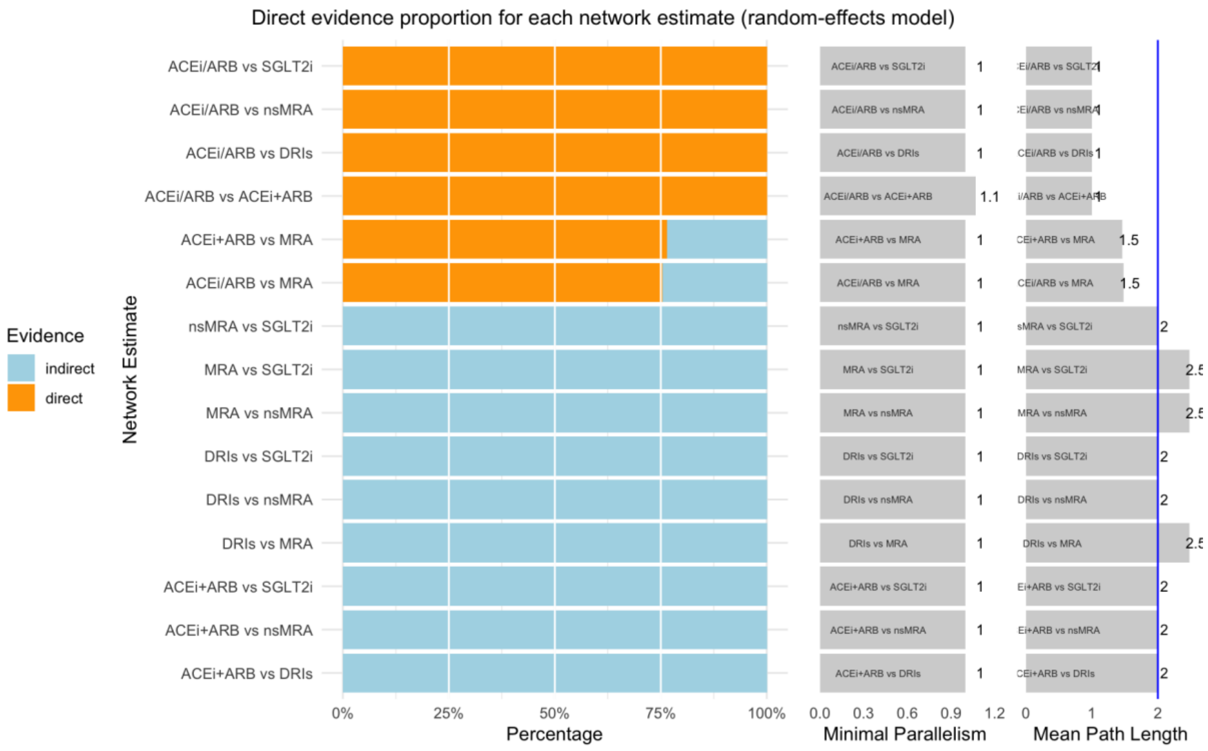

Hyperkalemia

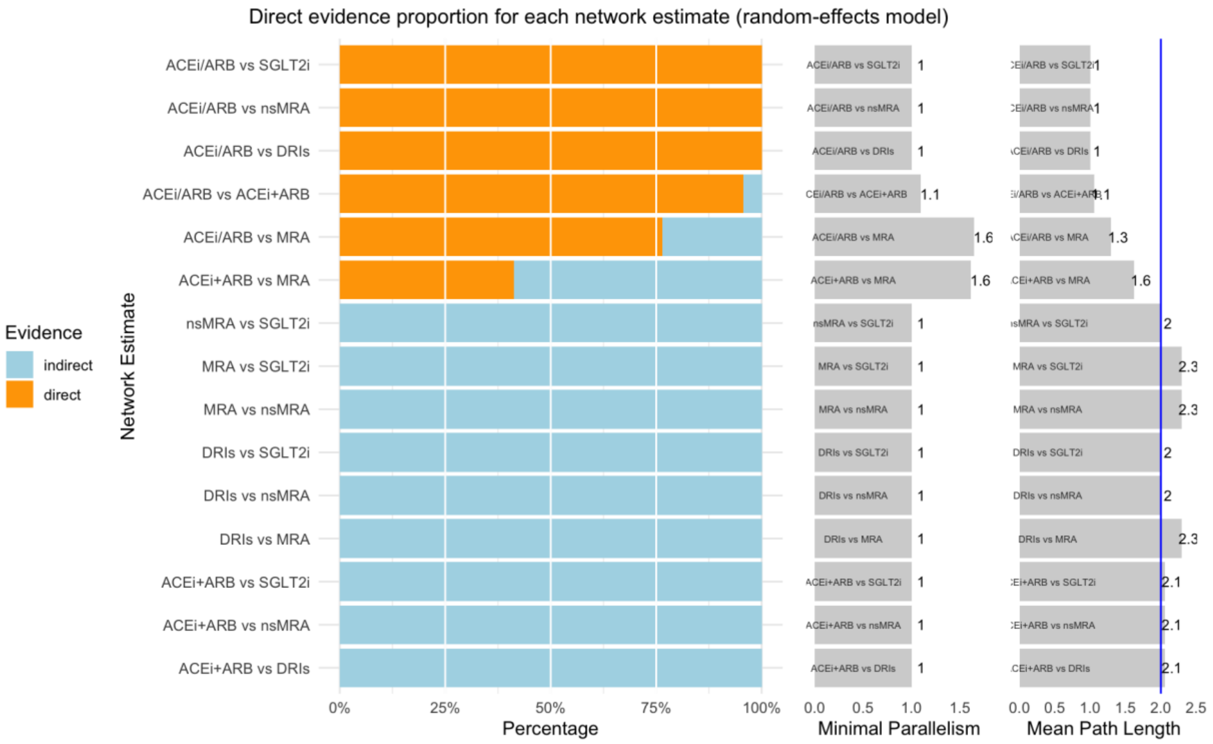

Hypotension

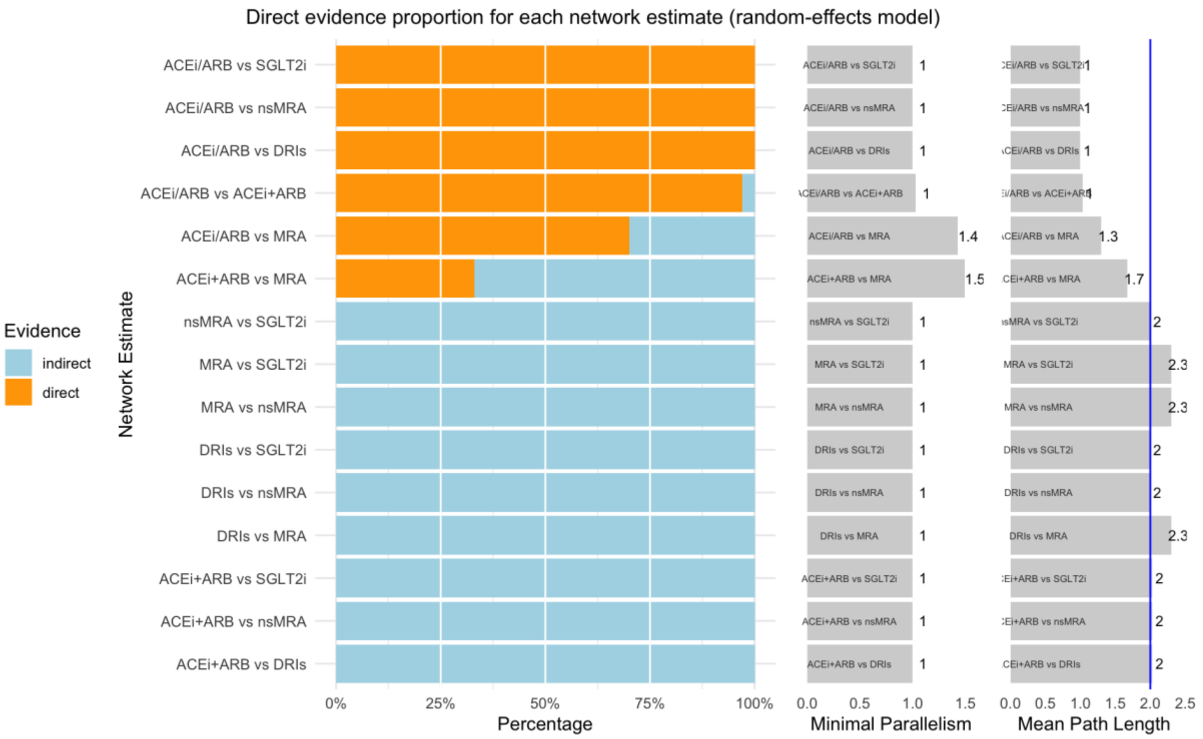

Supplement: S6 File — (PDF) [file pone.0293183.s006.pdf]
