# Supplementary material for: Treatment of diabetic kidney disease. A network meta-analysis
Source: PLoS One. 2023 Nov 2;18(11):e0293183. doi: 10.1371/journal.pone.0293183 (PMC10621862; doi:10.1371/journal.pone.0293183)
Supplement: S7 File — (PDF) [file pone.0293183.s007.pdf]

S7 GRADE protocol

1. **Study limitations**:
    - downgrade (-1, serious concerns) when contribution from low comparisons (less than 30%) or if moderate/high RoB contributed more than 70%
    - downgrade (-2, very serious concerns) contributions from high RoB comparisons (>70%)

2. **Imprecision**:
    - downgrading (-1) for a wide 95% confidence intervals, whereby the clinical decision would be clearly different for the lowest or highest boundary of the 95% CI
    - downgrade (-2) for close to null effect
    - if direct evidence was already downgraded due to imprecision, the resulting indirect evidence was not downgraded again

3. **Inconsistency**: integrating Inconsistency and heterogeneity
   Inconsistency: variation in treatment estimates between designs: Hence we separated direct from indirect evidence (SIDE) using the back-calculation method.
    - Downgrade (-1) for inconsistency (if p<0,30**???** for difference between direct and indirect evidence) or overall heterogeneity (I2) was >50% or point estimates/95% CI vary widely
    - Downgrade (-1) not possible to assess due to lack of direct evidence

4. **Indirectness**:
    - Downgrade for intransitivity (-1) in case of a narrow study population (e.g. only patients with CKD stage 3),
    - We ensured transitivity by including only patients with Diabetes, CKD and with regards to the interventions including only studies with a basic single RAAS inhibition (>80%) on top of the actual intervention. To address studies, that are included due to the arbitrary determined cut off level of a minimum of 80% single ACEi/ARB, we conducted a sensitivity analysis excluding such studies. The robustness of our results was reassured.

5. **Publication bias**: We applied the comparison-adjusted funnel plot for detecting any bias (interventions arranged from oldest to newest)
    - If a relevant publication bias was detected (-1)

In case of a simultaneous contributions of direct and indirect evidence we chose the higher of the two confiedence ratings for the NMA estimate. But in situations where one contribution (indirect vs. direct) is clearly more precise, we favored the quality rating of the more precise one.
With regards to indirect comparisons, we mainly focused on first order loops. If the NMA comparison only consists of indirect comparisons, our initial rating starts from the lowest confidence rating of the contributing direct evidence and will eventually be further downgraded according to our criteria.
