# Supplementary material for: Treatment of diabetic kidney disease. A network meta-analysis
Source: PLoS One. 2023 Nov 2;18(11):e0293183. doi: 10.1371/journal.pone.0293183 (PMC10621862; doi:10.1371/journal.pone.0293183)

S8 splitting into direct and indirect evidence

Overall mortality

Comparison	Number of Studies	Direct Evidence	OR	95%-CI
**ACEi+ARB vs ACEi/ARB**				
Direct estimate	5	1.00	0.98	[0.72; 1.34]
Indirect estimate				
Network estimate			0.98	[0.72; 1.34]
**DRIs vs ACEi/ARB**				
Direct estimate	2	1.00	1.05	[0.82; 1.34]
Indirect estimate				
Network estimate			1.05	[0.82; 1.34]
**MRA vs ACEi/ARB**				
Direct estimate	1	1.00	0.22	[0.01; 4.94]
Indirect estimate				
Network estimate			0.22	[0.01; 4.94]
**nsMRA vs ACEi/ARB**				
Direct estimate	4	1.00	0.89	[0.74; 1.07]
Indirect estimate				
Network estimate			0.89	[0.74; 1.07]
**SGLT2i vs ACEi/ARB**				
Direct estimate	6	1.00	0.81	[0.70; 0.95]
Indirect estimate				
Network estimate			0.81	[0.70; 0.95]
**ACEi+ARB vs DRIs**				
Direct estimate	0	0		
Indirect estimate			0.94	[0.63; 1.39]
Network estimate			0.94	[0.63; 1.39]
**ACEi+ARB vs MRA**				
Direct estimate	0	0		
Indirect estimate			4.37	[0.20; 97.62]
Network estimate			4.37	[0.20; 97.62]
**ACEi+ARB vs nsMRA**				
Direct estimate	0	0		
Indirect estimate			1.10	[0.77; 1.58]
Network estimate			1.10	[0.77; 1.58]
**ACEi+ARB vs SGLT2i**				
Direct estimate	0	0		
Indirect estimate			1.21	[0.86; 1.71]
Network estimate			1.21	[0.86; 1.71]
**DRIs vs MRA**				
Direct estimate	0	0		
Indirect estimate			4.66	[0.21; 103.60]
Network estimate			4.66	[0.21; 103.60]
**DRIs vs nsMRA**				
Direct estimate	0	0		
Indirect estimate			1.18	[0.87; 1.60]
Network estimate			1.18	[0.87; 1.60]
**DRIs vs SGLT2i**				
Direct estimate	0	0		
Indirect estimate			1.29	[0.97; 1.72]
Network estimate			1.29	[0.97; 1.72]
**MRA vs nsMRA**				
Direct estimate	0	0		
Indirect estimate			0.25	[0.01; 5.59]
Network estimate			0.25	[0.01; 5.59]
**MRA vs SGLT2i**				
Direct estimate	0	0		
Indirect estimate			0.28	[0.01; 6.10]
Network estimate			0.28	[0.01; 6.10]
**nsMRA vs SGLT2i**				
Direct estimate	0	0		
Indirect estimate			1.09	[0.86; 1.39]
Network estimate			1.09	[0.86; 1.39]

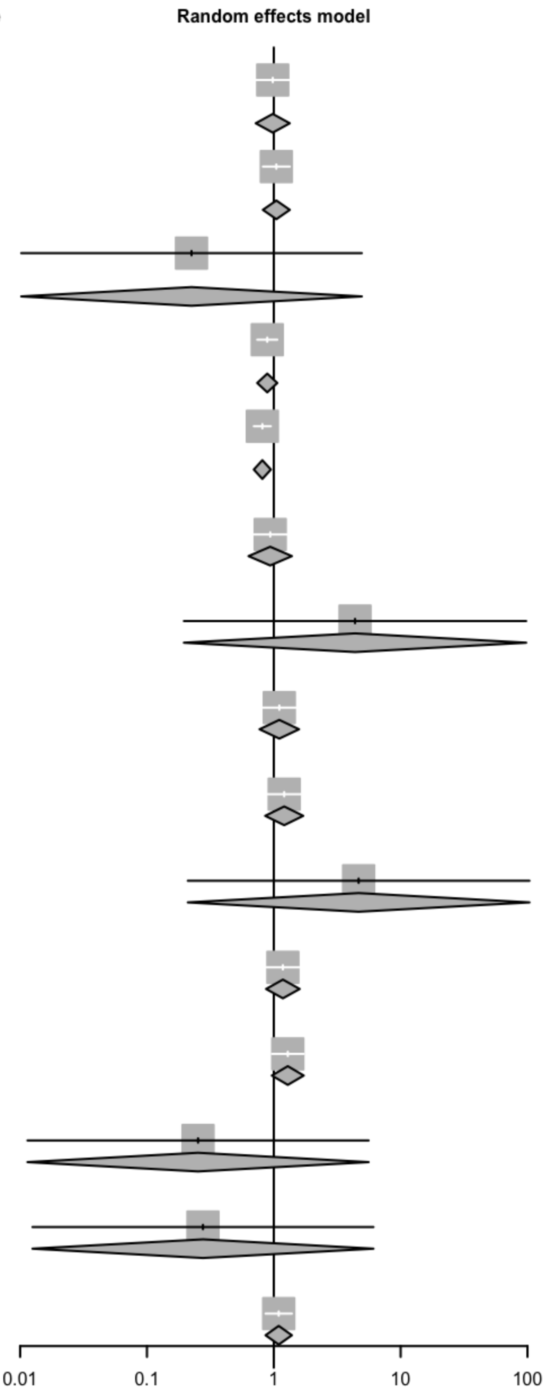

End stage kidney disease

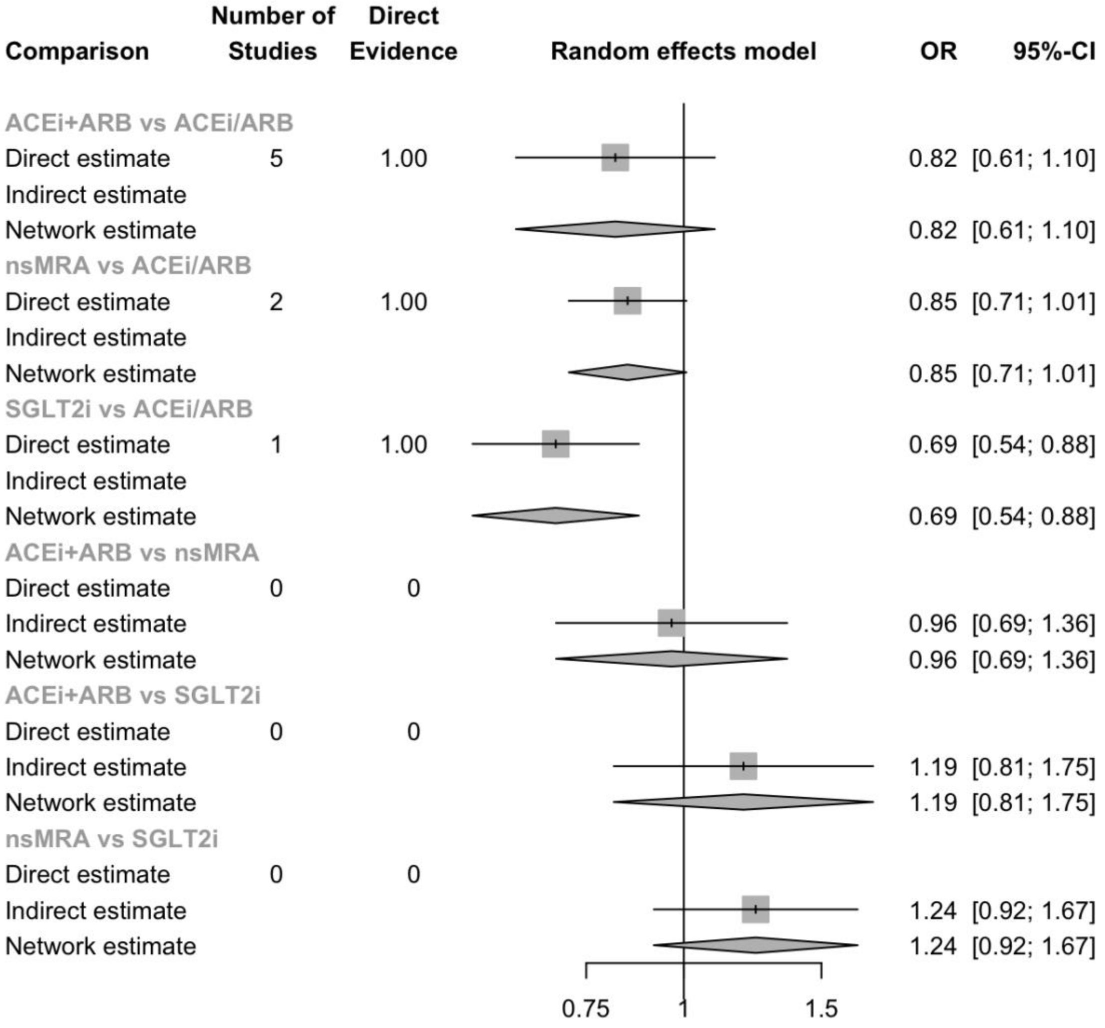

renal composite outcome

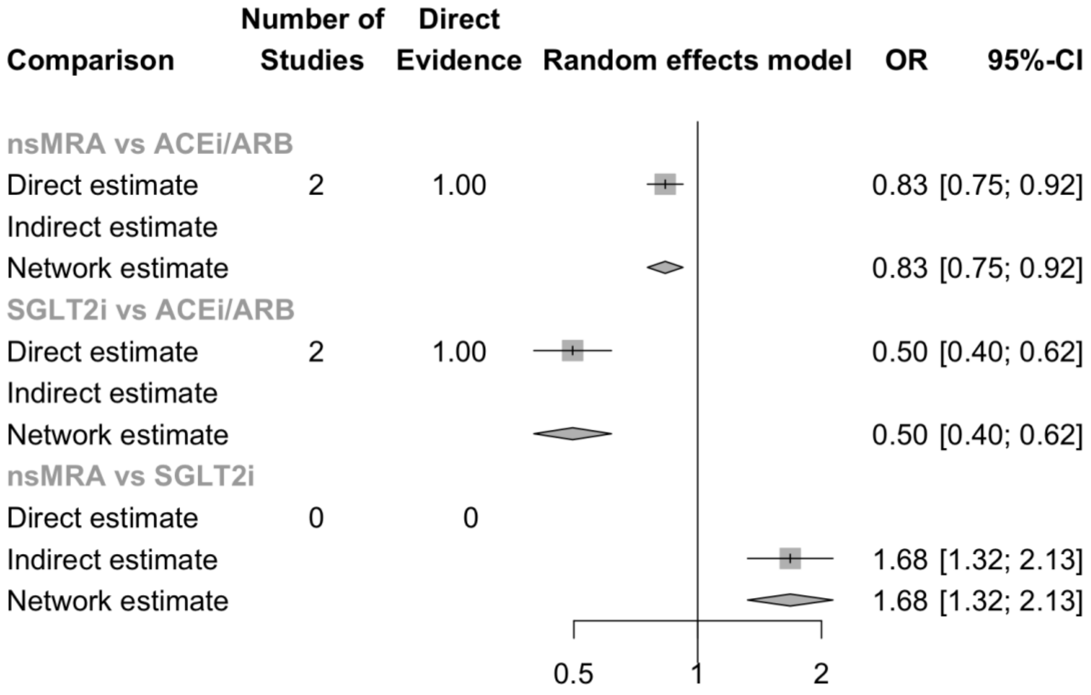

Hypotension

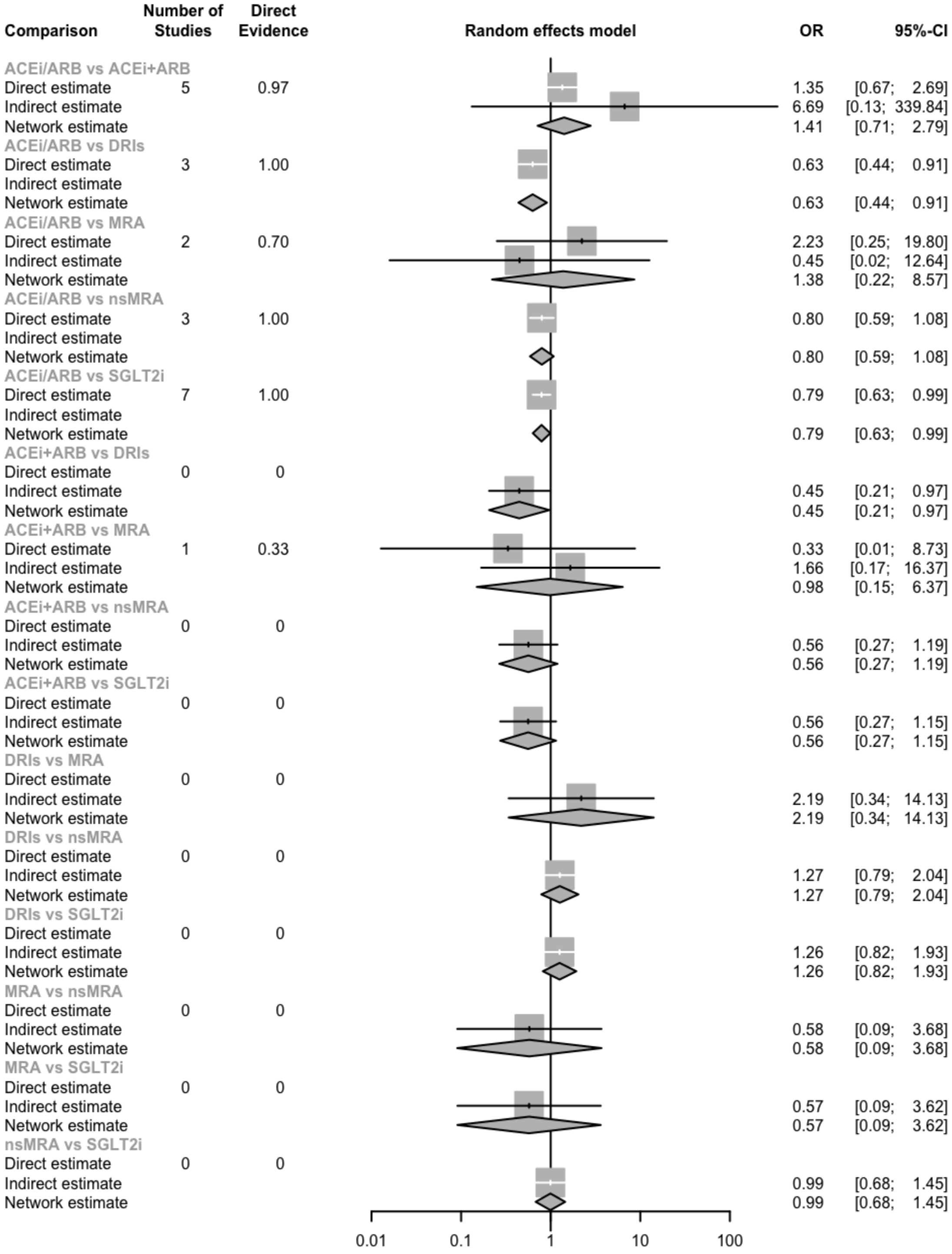

Acute kidney injury

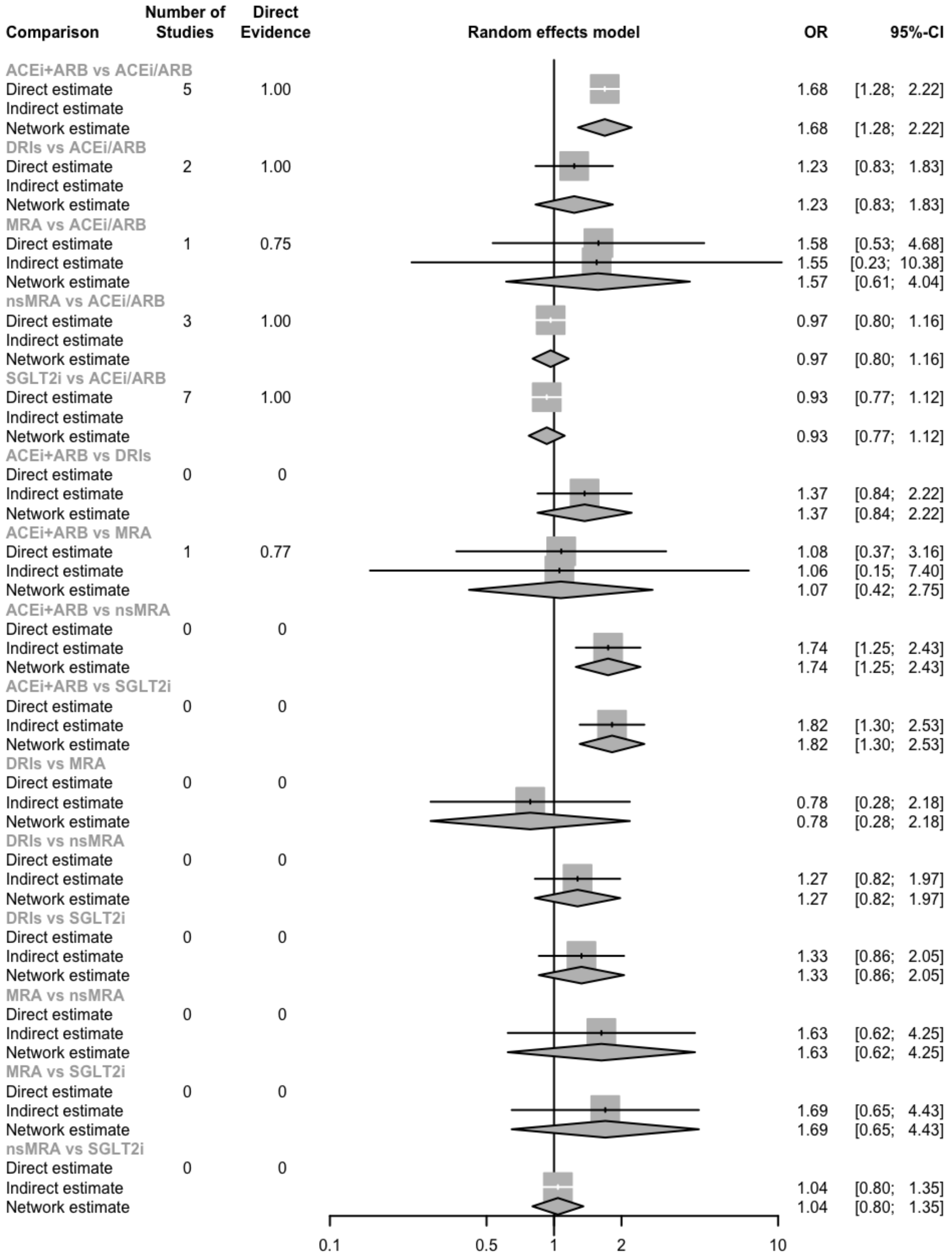

Hyperkalemia

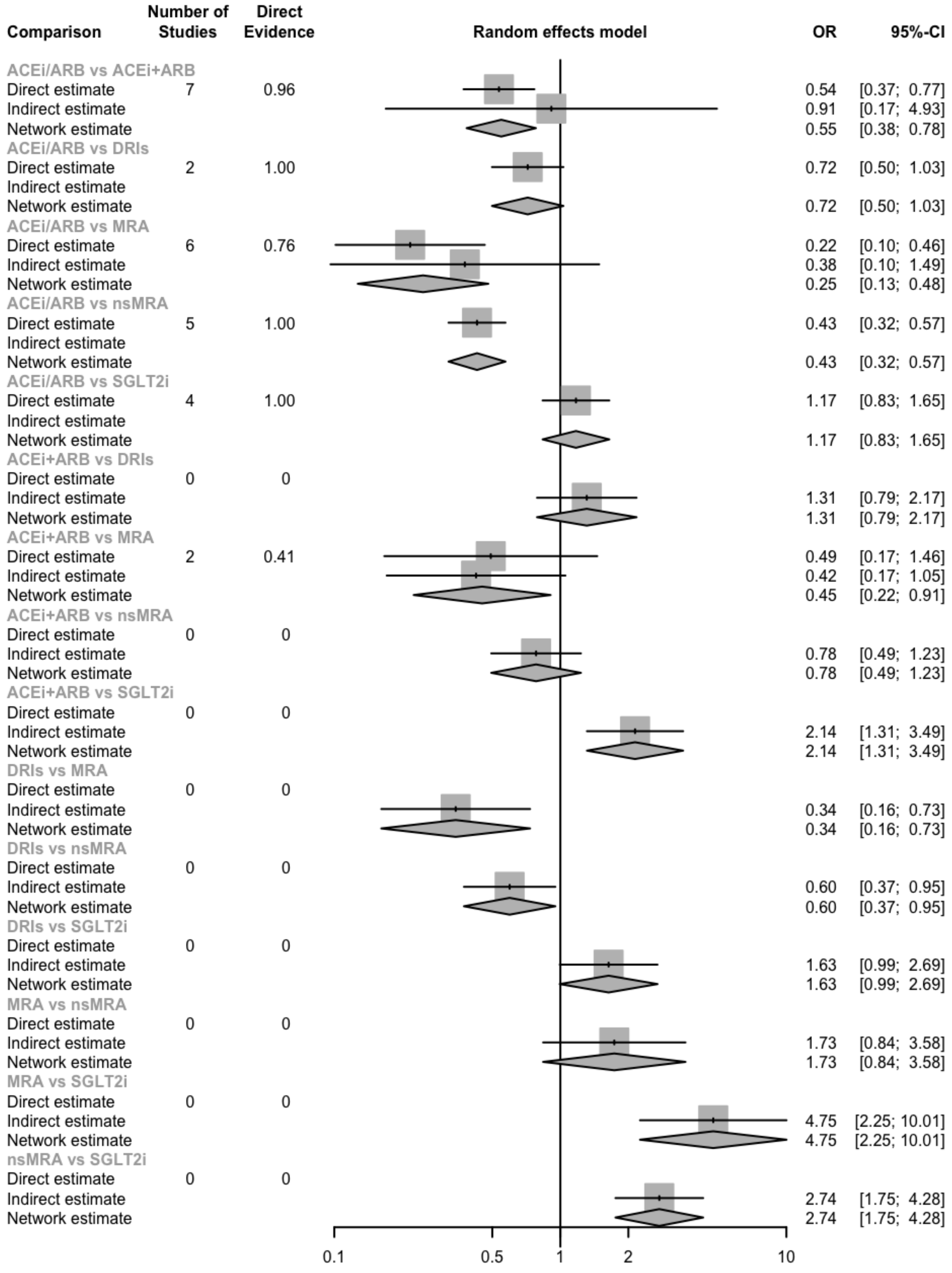

Supplement: S8 File — (PDF) [file pone.0293183.s008.pdf]
