# Supplementary material for: Treatment of diabetic kidney disease. A network meta-analysis
Source: PLoS One. 2023 Nov 2;18(11):e0293183. doi: 10.1371/journal.pone.0293183 (PMC10621862; doi:10.1371/journal.pone.0293183)
Supplement: S9 File — (PDF) [file pone.0293183.s009.pdf]

S9 Risk of bias

Unique ID	Study ID	Experimental	Comparator	Outcome	D1	D2	D3	D4	D5	Overall
Morgensen 20(	Morgensen 2000	ACEi+ARB	singleRAASi	Albuminuria, Hypotension	+	+	+	+	+	+
Tütüncü 2001	Tütüncü 2001	ACEi+ARB	singleRAASi	Albuminuria, Hypotension	!	!	+	+	+	!
Nakamura 200:	Nakamura 2002	ACEi+ARB	singleRAASi	NA	-	+	+	+	!	-
Cetinkaya 2004	Cetinkaya 2004	ACEi+ARB	singleRAASi	NA	-	+	+	+	+	-
Atmaca 2006	Atmaca 2006	ACEi+ARB	singleRAASi	Albuminuria, Hypotension	!	+	+	+	+	!
Igarashi 2006	Igarashi 2006	ACEi+ARB	singleRAASi	NA	!	+	+	+	+	!
Ogawa 2007	Ogawa 2007	ACEi+ARB	singleRAASi	NA	+	+	+	+	+	+
Krairittichai 200	Krairittichai 2009	ACEi+ARB	singleRAASi	NA	!	+	+	+	+	!
Titan 2011	Titan 2011	ACEi+ARB	singleRAASi	NA	!	+	+	+	!	!
Fried 2013	Fried 2013	ACEi+ARB	singleRAASi	NA	+	+	+	!	!	!
Imai 2013	Imai 2013	ACEi+ARB	singleRAASi	NA	!	+	!	+	+	!
Fernandez 201:	Fernandez 2013	ACEi+ARB	singleRAASi	NA	+	+	+	+	+	+
Nakamura 201:	Nakamura 2013	ACEi+ARB	singleRAASi	NA	!	+	+	+	+	!
Saglimbene 20:	Saglimbene 2018	ACEi+ARB	singleRAASi	NA	+	+	+	+	+	+
Ruggeneti 2019	Ruggeneti 2019	ACEi+ARB	single RAASi	NA	+	+	!	+	+	!
Zhang 2019	Zhang 2019	ACEi+ARB	singleRAASi	NA	!	+	+	!	+	!
Persson 2010	Persson 2010	DRI	singleRAASi	NA	+	+	+	+	+	+
Bakris 2013	Bakris 2013	DRI	singleRAASi	NA	+	+	+	-	+	-
Soji 2015	Soji 2015	DRI	single RAASi	NA	!	+	!	+	!	!
ALTITUDE	Parving 2012	DRI	singleRAASi	NA	!	+	+	+	+	!
van den Meirac	van den Meiracker :	MRA	singleRAASi	NA	+	+	+	+	+	+
Epstein 2006	Epstein 2006	MRA	singleRAASi	NA	+	+	+	+	+	+
Epstein 2002	Epstein 2002	MRA	singleRAASi	NA	+	+	+	+	+	!
Ziaee 2013	Ziaee 2013	MRA	singleRAASi	NA	!	+	+	+	+	!
Kato 2015	Kato 2015	MRA	singleRAASi	NA	!	+	+	+	+	!
Chen 2018	Chen 2018	MRA	singleRAASi	NA	!	+	+	+	+	!
El Mokadem 2(	El Mokadem 2020	MRA	singleRAASi	NA	+	+	+	+	+	+
Yale 2014	Yale 2014	SGLT2i	singleRAASi	NA	+	+	+	+	+	+
Wanner 2020	Wanner 2020	SGLT2i	singleRAASi	NA	+	+	+	-	+	-
Fioretto 2018	Fioretto 2018	SGLT2i	singleRAASi	NA	+	+	+	+	+	+
Neuen 2019	Neuen 2019	SGLT2i	singleRAASi	NA	+	+	+	-	+	-
Perkovic 2019	Perkovic 2019	SGLT2i	singleRAASi	NA	+	+	+	+	+	+
Pollock 2019	Pollock 2019	SGLT2i	single RAASi	NA	+	+	+	+	+	+

Bhatt 2020	Bhatt 2020	SGLT2i	single RAASi	NA	+	+	+	+	+	+
Mosenzon 202(	Mosenzon 2020	SGLT2i	singleRAASi	NA	+	+	+	+	+	+
Heerspink 2020	Heerspink 2020	SGLT2i	singleRAASi	NA	+	+	+	+	+	+
Bakris 2015	Bakris 2015	nsMRA	singleRAASi	NA	+	+	+	+	+	+
Katayama 201€	Katayama 2016	nsMRA	singleRAASi	NA	+	+	+	+	+	+
Ito 2019	Ito 2019	nsMRA	singleRAASi	NA	+	+	+	+	+	+
Ito 2020	Ito 2020	nsMRA	singleRAASi	NA	+	+	+	+	+	+
Bakris 2020	Bakris 2020	nsMRA	single RAASi	NA	+	+	+	+	+	+
Pitt 2021	Pitt 2021	nsMRA	singleRAASi	NA	+	+	+	+	+	+
Mehdi 2009	Mehdi 2009	ACEi+ARB	MRA	NA	+	+	+	+	+	+
Esteghamati 20	Esteghamati 2013	ACEi+ARB	MRA	NA	!	+	!	+	+	!
Imbalzano 201!	Imbalzano 2015	ACEi+ARB	DRI	NA	!	+	+	+	+	!

+ Low risk

! Some concerns

- High risk

D1 Randomisation process

D2 Deviations from the intended interventions

D3 Missing outcome data

D4 Measurement of the outcome

D5 Selection of the reported result
