# Supplementary material for: Treatment of diabetic kidney disease. A network meta-analysis
Source: PLoS One. 2023 Nov 2;18(11):e0293183. doi: 10.1371/journal.pone.0293183 (PMC10621862; doi:10.1371/journal.pone.0293183)
Supplement: S11 File — (PDF) [file pone.0293183.s011.pdf]

S11 Sensitivity analysis (single ACEi/ARB mandatory in control groups)

Overall mortality (including only studies with single ACEi/ARB mandatory in control groups)

ACEi+ARB 0.9878 [0.7437; 1.3122]
DRIs 0.1967 [0.0094; 4.1141]
MRA 0.2245 [0.0103; 4.9080]
nsMRA 0.8876 [0.7869; 1.0010]
SGLT2i 0.8259 [0.6671; 1.0224]

Overall mortality (including all studies)

ACEi+ARB 0.9811 [0.7205; 1.3360]
DRIs 1.0472 [0.8202; 1.3371]
MRA 0.2245 [0.0102; 4.9377]
nsMRA 0.8880 [0.7400; 1.0656]
SGLT2i 0.8123 [0.6958; 0.9483]

End stage kidney disease
