# Supplementary material for: Treatment of diabetic kidney disease. A network meta-analysis
Source: PLoS One. 2023 Nov 2;18(11):e0293183. doi: 10.1371/journal.pone.0293183 (PMC10621862; doi:10.1371/journal.pone.0293183)

S12 Sensitivity analysis (with/without Fidelity)

Supplemental figure 12 a OR of mortality with using FIDELITY as a single study

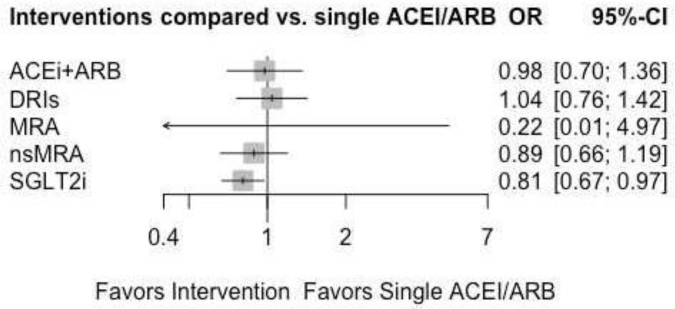

Supplemental figure 12 b OR of ESKD with using FIDELITY as a single study

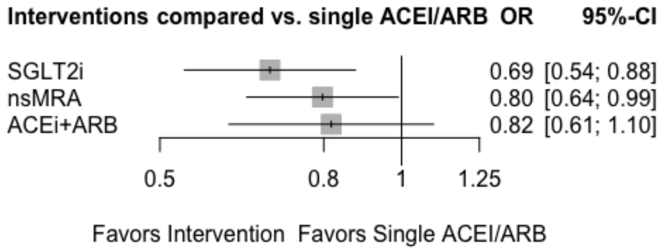

Supplement: S12 File — (PDF) [file pone.0293183.s012.pdf]
