# Supplementary material for: Treatment of diabetic kidney disease. A network meta-analysis
Source: PLoS One. 2023 Nov 2;18(11):e0293183. doi: 10.1371/journal.pone.0293183 (PMC10621862; doi:10.1371/journal.pone.0293183)
Supplement: S13 File — (PDF) [file pone.0293183.s013.pdf]

S13 Albuminuria

Albuminuria

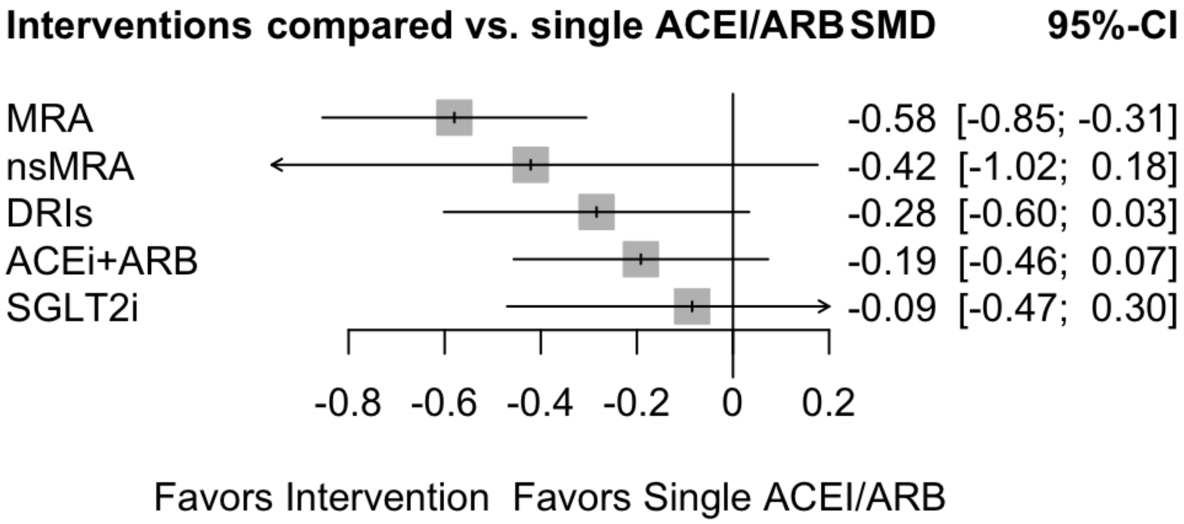

Figure S13: Albuminuria

OR = Odds ratio, 95%- CI = 95% Confidence interval, SMD= standardized mean difference, single ACEi/ARB= single Angiotensin-converting enzyme inhibitors or Angiotensin receptor blocker, ACEi+ARB = Angiotensin-converting enzyme inhibitors and Angiotensin receptor blocker combination, DRI= direct renin inhibitors, MRA= Mineralocorticoid receptor antagonists, nsMRA= non-steroidal Mineralocorticoid receptor antagonists, SGLT2i= Sodium glucose transporter inhibitors
