# Supplementary material for: Treatment of diabetic kidney disease. A network meta-analysis
Source: PLoS One. 2023 Nov 2;18(11):e0293183. doi: 10.1371/journal.pone.0293183 (PMC10621862; doi:10.1371/journal.pone.0293183)
Supplement: S15 File — (PDF) [file pone.0293183.s015.pdf]

<u>S15 R-code</u>

```
library(netmeta)
library(meta)
library("readxl")
data1<-read_excel("/Users/fbuettner/Desktop/Promo - Meta/R data/ESKD/ESKD.xlsx", 2)

#convert Data from Excel into a functioning R data frame

study<-data1$Study
e_exp<-as.numeric(data1$e_exp)
n_exp<-as.numeric(data1$n_exp)
e_con<-as.numeric(data1$e_con)
n_con<-as.numeric(data1$n_con)
treat1<-data1$`treat 1`
treat2<-data1$`treat 2`
newData<-data.frame(study, e_exp,n_exp, e_con, n_con, treat1, treat2)
newData
# Transform data from arm-based format to contrast-based format
p2 <- pairwise(treat = list(treat1, treat2),event = list(e_exp, e_con),n =
list(n_exp,n_con),data=newData,sm="OR")

#  Perform network.-Meta-analysis Random effect model
net2 <- netmeta(TE, seTE, treat1, treat2, studlab, data = p2, sm ="OR",comb.fixed = FALSE, comb.random
= TRUE,reference.group = "ACEi/ARB",details.chkmultiarm = TRUE,sep.trts = " vs ")
summary(net2)
#network graph
#edges
netgraph(net2, points=TRUE, cex.points=4, cex=2,number.of.studies =TRUE, scale = 0.9)
# Alterantive
netgraph(net2, points=TRUE, cex.points=4, cex=2,number.of.studies =TRUE, scale = 0.9, plastic = FALSE,
col="black")
#Visualizing Direct and Indirect Evidence
library("ggplot2")
library("gridExtra")
library(dmetar)
d.evidence <- direct.evidence.plot(net2, random=TRUE)
plot(d.evidence)

#Effect Estimate Table
result.matrix <-net2$TE.random
result.matrix <- round(result.matrix, 2)
result.matrix[lower.tri(result.matrix, diag = FALSE)] <- NA
result.matrix



lower_confidence <-net2$lower.random
lower_confidence <- round(result.matrix, 2)
lower_confidence[lower.tri(result.matrix, diag = FALSE)] <- NA
lower_confidence

upper_confidence <- net2$upper.random
```

```
upper_confidence <- round(result.matrix, 2)
upper_confidence[lower.tri(result.matrix, diag = FALSE)] <- NA
upper_confidence

#Treatment Ranking
netrank(net2, small.values = "good")
#Forest plot
forest(net2,sortvar = TE, xlim=c(0.50,1.25),reference.group="ACEi/ARB",leftlabs="Interventions",
      xlab="Favors Intervention  Favors Single ACEI/ARB",drop.reference.group=TRUE,
      smlab=paste(" compared vs. single ACEI/ARB"), pooled="random")

#Net heat plot
netheat(net2, random = TRUE)
netsplit(net2)
netsplit(net2)  %>% forest(show = "all")

#splitting direct indirect evidence
projectX<-netsplit(net2)
print(projectX)
forest(projectX, fontsize = 6, spacing = 0.5, addrow.subgroups = FALSE)
forest(projectX, fontsize = 6, spacing = 0.5, addrow.subgroups = FALSE, show = "with.direct")
forest(projectX, fontsize = 10, spacing = 1, addrow.subgroups = FALSE, show = "all")
forest(projectX)

#Comparison-Adjusted Funnel Plots
funnel(net2,
      order = c("ACEi/ARB","ACEi+ARB","SGLT2i", "nsMRA"), pch = c(1:3),
      col = c("blue", "red", "forestgreen"),
      linreg = TRUE)
```
