# Supplementary material for: Treatment of diabetic kidney disease. A network meta-analysis
Source: PLoS One. 2023 Nov 2;18(11):e0293183. doi: 10.1371/journal.pone.0293183 (PMC10621862; doi:10.1371/journal.pone.0293183)
Supplement: S16 File — (PDF) [file pone.0293183.s016.pdf]

S16 Data sheets

AKI

**Study**	e_exp	**n_exp**	e_con	**n_con**	**e_thirdA**	**n_thirdA**	treat 1	treat 2	treat 3
Titan 2011	1	28	1	28	NA	NA	ACEi+ARB	ACEi/ARB	NA
VA NEPHRON-D	130	724	80	724	NA	NA	ACEi+ARB	ACEi/ARB	NA
ORIENT 2013	0	205	1	209	NA	NA	ACEi+ARB	ACEi/ARB	NA
Nakamura 2013	0	14	0	14	NA	NA	ACEi+ARB	ACEi/ARB	NA
VALID	8	33	14	70	NA	NA	ACEi+ARB	ACEi/ARB	NA
AVOID	2	301	0	298	NA	NA	DRIs	ACEi/ARB	NA
ALTITUDE	53	3137	45	3190	NA	NA	DRIs	ACEi/ARB	NA
Yale 2014	1	89	1	90	NA	NA	SGLT2i	ACEi/ARB	NA
EMPA-REG Outcome 2015	8	509	6	260	NA	NA	SGLT2i	ACEi/ARB	NA
Derive Study	1	160	2	161	NA	NA	SGLT2i	ACEi/ARB	NA
Canvas	17	1728	17	1298	NA	NA	SGLT2i	ACEi/ARB	NA
Credence	86	2202	98	2199	NA	NA	SGLT2i	ACEi/ARB	NA
Delight	0	145	2	148	NA	NA	SGLT2i	ACEi/ARB	NA
SCORED	116	5292	111	5292	NA	NA	SGLT2i	ACEi/ARB	NA
Ito 2019	0	70	0	73	NA	NA	nsMRA	ACEi/ARB	NA
ESAX-DN	12	222	5	227	NA	NA	nsMRA	ACEi/ARB	NA
FIDELIO-DKD	129	2833	136	2841	NA	NA	nsMRA	ACEi/ARB	NA
Mehdi 2009	13	26	10	27	13	27	ACEi+ARB	ACEi/ARB	MRA
Figaro DKD	91	3686	98	3666	NA	NA	nsMRA	ACEi/ARB	NA

Overall mortality

Study	e_exp	n_exp	e_con	n_con	treat 1	treat 2
VA NEPHRON-D	63	724	60	724	ACEi+ARB	ACEi/ARB
ORIENT 2013	14	205	16	209	ACEi+ARB	ACEi/ARB
PRONEDI 2013	6	70	3	63	ACEi+ARB	ACEi/ARB
Nakamura 2013	0	14	0	14	ACEi+ARB	ACEi/ARB
LIRICO	15	355	33	704	ACEi+ARB	ACEi/ARB
VALID	1	33	9	70	ACEi+ARB	ACEi/ARB
ALTITUDE	376	4274	358	4287	DRIs	**ACEi/ARB**
AVOID	0	301	2	298	DRIs	ACEi/ARB
van den Meiracker 2006	0	24	2	29	MRA	ACEi/ARB
Yale 2014	0	90	2	90	SGLT2i	ACEi/ARB
EMPA-REG Outcome 2015	63	509	41	260	SGLT2i	ACEi/ARB
Derive Study	0	160	0	161	SGLT2i	ACEi/ARB
Canvas	140	1728	160	1298	SGLT2i	ACEi/ARB
Credence	168	2202	201	2199	SGLT2i	ACEi/ARB
Delight	1	145	0	148	SGLT2i	ACEi/ARB
SCORED	246	5292	246	5292	SGLT2i	ACEi/ARB
ARTS - DN	0	117	1	94	nsMRA	ACEi/ARB
ARTS-DN Japan	0	12	0	12	nsMRA	ACEi/ARB
ESAX-DN	1	222	0	227	nsMRA	ACEi/ARB
FIDELIO-DKD	219	2833	244	2841	nsMRA	ACEi/ARB
Figaro DKD	333	3686	370	3666	nsMRA	ACEi/ARB

ESKD

**Study**	e_exp	**n_exp**	e_con	**n_con**	treat 1	treat 2
VA NEPHRON-D	27	724	43	724	ACEi+ARB	ACEi/ARB
ORIENT 2013	48	205	49	209	ACEi+ARB	ACEi/ARB
PRONEDI 2013	10	70	11	63	ACEi+ARB	ACEi/ARB
LIRICO	2	355	7	704	ACEi+ARB	ACEi/ARB
VALID	9	33	17	70	ACEi+ARB	ACEi/ARB
Credence	116	2202	165	2199	SGLT2i	ACEi/ARB
FIDELIO-DKD	208	2833	235	2841	nsMRA	ACEi/ARB
Figaro DKD 2021	46	3686	62	3666	nsMRA	ACEi/ARB

Composite outcome

Study	e_exp	n_exp	e_con	n_con	treat 1	treat 2
Canvas	66	1728	92	1298	SGLT2i	ACEi/ARB
Declare Timi 58	70	2611	141	2588	SGLT2i	ACEi/ARB
	504	2833	600	2841		
FIDELIO-DKD					nsMRA	ACEi/ARB
Figaro DKD	350	3686	395	3666	nsMRA	ACEi/ARB

Hyperkalemia

Study	e_exp	n_exp	e_con	n_con	e_thirdA	n_thirdA	treat 1	treat 2	treat 3
Titan 2011	3	28	1	28	NA	NA	ACEi+ARB	ACEi/ARB	NA
VA NEPHRON-D	98	724	41	724	NA	NA	ACEi+ARB	ACEi/ARB	NA
ORIENT 2013	24	205	15	209	NA	NA	ACEi+ARB	ACEi/ARB	NA
PRONEDI 2013	8	70	7	63	NA	NA	ACEi+ARB	ACEi/ARB	NA
Nakamura 2013	0	14	0	14	NA	NA	ACEi+ARB	ACEi/ARB	NA
LIRICO	7	355	11	704	NA	NA	ACEi+ARB	ACEi/ARB	NA
VALID	3	33	13	70	NA	NA	ACEi+ARB	ACEi/ARB	NA
AVOID	15	301	17	298	NA	NA	DRIs	ACEi/ARB	NA
ViVID	0	80	0	65	NA	NA	DRIs	ACEi/ARB	NA
ALTITUDE	1670	4272	1244	4285	NA	NA	DRIs	ACEi/ARB	NA
van den Meiracker 2006	5	29	1	30	NA	NA	MRA	ACEi/ARB	NA
Kato 2015	0	26	0	26	NA	NA	MRA	ACEi/ARB	NA
Chen 2018	6	55	1	54	NA	NA	MRA	ACEi/ARB	NA
El Mokadem 2020	2	25	1	25	NA	NA	MRA	ACEi/ARB	NA
Yale 2014	29	89	18	90	NA	NA	SGLT2i	ACEi/ARB	NA
EMPA-REG Outcome 2015	16	509	19	260	NA	NA	SGLT2i	ACEi/ARB	NA
Canvas	6	1728	5	1298	NA	NA	SGLT2i	ACEi/ARB	NA
Credence	151	2200	181	2197	NA	NA	SGLT2i	ACEi/ARB	NA
ARTS - DN	2	117	0	94	NA	NA	nsMRA	ACEi/ARB	NA
Ito 2019	2	70	1	73	NA	NA	nsMRA	ACEi/ARB	NA
ESAX-DN	20	222	5	227	NA	NA	nsMRA	ACEi/ARB	NA
FIDELIO-DKD	516	2833	255	2841	NA	NA	nsMRA	ACEi/ARB	NA
Mehdi 2009	10	26	14	27	2	27	ACEi+ARB	MRA	ACEi/ARB
Esteghamati 2013	0	62	3	74	NA	NA	ACEi+ARB	MRA	NA
Epstein 2006	5	82	3	88	NA	NA	MRA	ACEi/ARB	NA

Epstein 2002	8	67	2	74	NA	NA	MRA	ACEi/ARB	NA
Figaro DKD	396	3686	193	3666	NA	NA	nsMRA	ACEi/ARB	NA

Hypotension

**Study**	e_exp	**n_exp**	e_con	**n_con**	**e_thirdA**	**n_thirdA**	treat 1	treat 2	treat 3
CALM	1	67	4	130	NA	NA	ACEi+ARB	ACEi/ARB	NA
Tütüncü 2001	0	10	0	24	NA	NA	ACEi+ARB	ACEi/ARB	NA
Atmaca 2006	0	8	0	18	NA	NA	ACEi+ARB	ACEi/ARB	NA
Ogawa 2007	1	72	1	74	NA	NA	ACEi+ARB	ACEi/ARB	NA
VA NEPHRON-D	12	724	14	724	NA	NA	ACEi+ARB	ACEi/ARB	NA
ORIENT 2013	0	205	0	209	NA	NA	ACEi+ARB	ACEi/ARB	NA
LIRICO	1	355	5	704	NA	NA	ACEi+ARB	ACEi/ARB	NA
VALID	1	33	3	70	NA	NA	ACEi+ARB	ACEi/ARB	NA
AVOID	12	301	3	298	NA	NA	DRIs	ACEi/ARB	NA
ALTITUDE	519	4272	357	4285	NA	NA	DRIs	ACEi/ARB	NA
ViVID	0	80	2	65	NA	NA	DRIs	ACEi/ARB	NA
El Mokadem 2020	1	22	0	23	NA	NA	MRA	ACEi/ARB	NA
Yale 2014	16	179	5	90	NA	NA	SGLT2i	ACEi/ARB	NA
EMPA-REG Outcome 2015	30	509	12	260	NA	NA	SGLT2i	ACEi/ARB	NA
Derive Study	3	160	0	161	NA	NA	SGLT2i	ACEi/ARB	NA
Canvas	165	1728	114	1298	NA	NA	SGLT2i	ACEi/ARB	NA
Credence	144	2202	115	2199	NA	NA	SGLT2i	ACEi/ARB	NA
Delight	4	145	4	148	NA	NA	SGLT2i	ACEi/ARB	NA
SCORED	281	5292	212	5292	NA	NA	SGLT2i	ACEi/ARB	NA
ARTS - DN	1	117	2	94	NA	NA	nsMRA	ACEi/ARB	NA
Ito 2019	0	70	0	73	NA	NA	nsMRA	ACEi/ARB	NA
FIDELIO-DKD	146	2833	153	2841	NA	NA	nsMRA	ACEi/ARB	NA
Mehdi 2009	0	26	NA	NA	1	27	ACEi+ARB	ACEi/ARB	MRA

Epstein 2006	0	86	5	91	NA	NA	MRA	ACEi/ARB	NA
Figaro DKD	156	3686	90	3666	NA	NA	nsMRA	ACEi/ARB	NA
