# Supplementary material for: Treatment of diabetic kidney disease. A network meta-analysis
Source: PLoS One. 2023 Nov 2;18(11):e0293183. doi: 10.1371/journal.pone.0293183 (PMC10621862; doi:10.1371/journal.pone.0293183)
Supplement: S17 File — (PDF) [file pone.0293183.s017.pdf]

S 17 GRADE for renal composite outcome

**Renal composite outcome**

| **GRADE** | **final quality evaluation** |
|---|---|
| nsMRA vs ACEi/ARB | high |
| SGLT2i vs ACEi/ARB | high |
| nsMRA vs SGLT2i | moderate# |
