# Supplementary material for: Treatment of diabetic kidney disease. A network meta-analysis
Source: PLoS One. 2023 Nov 2;18(11):e0293183. doi: 10.1371/journal.pone.0293183 (PMC10621862; doi:10.1371/journal.pone.0293183)
Supplement: S18 File — (PDF) [file pone.0293183.s018.pdf]

S 18 GRADE for hypotension

Hypotension

GRADE	final quality evaluation
ACEi+ARB vs ACEi/ARB	moderate
DRIs vs ACEi/ARB	high
MRA vs ACEi/ARB	low
nsMRA vs ACEi/ARB	moderate
SGLT2i vs ACEi/ARB	high
ACEi+ARB vs DRIs	low"
ACEi+ARB vs MRA	low*
ACEi+ARB vs nsMRA	low"
ACEi+ARB vs SGLT2i	low"
DRIs vs MRA	very low*
DRIs vs nsMRA	low*
DRIs vs SGLT2i	moderate#
MRA vs nsMRA	very low*
MRA vs SGLT2i	very low*
nsMRA vs SGLT2i	low"
