# Supplementary material for: Treatment of diabetic kidney disease. A network meta-analysis
Source: PLoS One. 2023 Nov 2;18(11):e0293183. doi: 10.1371/journal.pone.0293183 (PMC10621862; doi:10.1371/journal.pone.0293183)
Supplement: Attachment — Submitted filename: Response to Reviewers.docx [file pone.0293183.s019.docx]

Dear editor,

We thank the reviewers for their constructive comments on our manuscript and have revised the manuscript based on theirs concerns.

We have addressed each point raised by the reviewers and believe the manuscript is now appropriate for publication in PLOS ONE.

**Reviewer #1**
*This is a reasonably well-done meta-analysis but the reality is that all clinical trials are not the same and go beyond randomization and double-blind protocols. One of the main distinctions between studies done with* ***NS-MRA and SGLT2 inhibitors is that maximal dosing of ACEI/ARB was NOT mandated in the SGLT2i studies****. I can assure you this was an issue not discussed with the design of the renal SGLT2 I trials. This will affect outcomes as has been shown in analyses evaluating lower doses of RAS blockade in people from those trials (Epstein, M., et al. Am J Manag Care 2015; 21(11 Suppl): S212-220; and data within the 2001 Brenner et.al. and Lewis et.al. NEJM papers).*

- We thank the reviewer for this important comment. Problems like this are common for most meta-analyses and may be the source of heterogeneity. Besides the SGLT2i study CREDENCE the great majority of SGTL2i studies did not have the requirement of using a maximum tolerated dose of ACEi/ARBs. This is one reason, why we hesitated to declare a superiority of SGLT2i above nsMRA. We added the following sentence in the limitations (page 21, first paragraph)“

Moreover, we could not consider different doses of each drug and differences in ACEi/ARB doses. In particular, most clinical trials including SGLT2i did not require the maximal labeled dose of ACEi/ARB. This however was the case in the studies including nsMRA (5). This hampers the comparability of these drug classes..”

*Moreover, many of the original trials in people with* ***eGFR above 45 were focused on glucose control and not renal outcomes****. The 3 trials CREDENCE, DAPA-CKD and Empa-KIDNEY did focus on appropriately powered renal and the secondary endpoint all-cause morality.*

- We are aware of the issue, that the majority of SGLT2i studies (besides CREDENCE, DAPA-CKD, EMPA-Kidney) did focus on glucose control, which may have resulted in exclusion of patient with severe kidney disease. We added a remark in our limitation section. (page 21 first paragraph). With reference to the statement to SGLT2i studies with eGFR >45: We absolutely agree to this point, which is also a problem many meta-analysis have to struggle with. Jardine, M. et al. 2020 conducted a secondary analysis of the CREDENCE trial and she found that the effect of SGLT2i was consistent across eGFR subgroups (30-45, 45-60, 60-90 ml/min per 1.73m2). But this analysis also stated that subgroups with lower eGFR exhibited a larger absolute benefit for renal outcomes. (page 21 first paragraph)

“In addition, SGLT2i studies mainly had renal secondary endpoints and mostly included patients with an eGFR above 45 mL/min/1.73 m^2^.”

*The statement about KDIGO guidelines is also not quite correct. The Am Diabetes Assoc guidelines 2023 is very clear that SGLT2 inhibitors are mandated if advanced CKD or heart failure is present irrespective of glycemic control and KDIGO is mirroring that, however, NS-MRA are also highly supported to be used with SGLT2 inhibitors is albuminuria is present as they have shown and the authors appropriately state protection against ESRD and reduction in heart failure hospitalization. Thus, like in heart failure,* ***all 3 are suggested*** *for treatment in this case and thus, the wording should be less absolute and imply that all 3 are needed if albuminuria is present which is what all studies of CKD were. Actually, the comment about guidelines should be excluded or include ADA guidelines with a less absolute statement to include both agents.*

- Thank you for pointing this out. We agree and changed our last paragraph (page 21) to:

“In conclusion, SGLT2i in our analysis was the only drug class showing beneficial effects on top of ACEi/ARB treatment regarding mortality and end stage kidney disease. nsMRA were based on less data, but diminished the risk for our renal composite outcome without safety concerns. Our data support the actual ADA and KDIGO guidelines, which suggest that all three drug classes (ACEi/ARB, SGLT2i and nsMRA) are needed in diabetic patients with albuminuria.”

**Reviewer #2**: 1. "**key learning points"**

*The statement "New clinical trials with SGLT2i and nsMRA emerged" is not accurate as clinical trials on these drug classes have been ongoing for several years.*

- Thank you for the advice. We changed the sentences to (page 4, paragraph 1)

“Clinical trials with SGLT2i and nsMRA demonstrated beneficial effects on various outcomes.”

*The statement "direct comparisons between these two drug classes and comparison to older drug classes/combination (MRA, DRI, ACEi+ARB combination) are missing" is incorrect as the study mentioned in the background section actually does include comparisons between these drug classes.*

- We apologize, but we could not identify the mentioned study. Could you please let us know, which study do you specifically mean? We are happy to add the changes.

*The statement "For MRA and DRI existed no sufficient hard outcome data" is incorrect as there have been clinical trials on these drug classes that have reported hard outcome data.*

- We agree, our statement is misleading. We rephrased this sentence (page 4, paragraph 2)

“For MRA and DRI existed no sufficient hard outcome data that met our inclusion criteria.”

*The statement "DRI in addition to ACEi/ARB or ACEi+ARB combinations are not recommended" is not accurate as some clinical guidelines do recommend the use of DRI in certain cases.*

*The statement "the usage of MRA is questionable due to missing hard endpoint data" is not entirely accurate as there are some clinical trials that have reported hard endpoint data on the use of MRA, although more data may be needed to fully assess their efficacy and safety.*

“Our data does not allow any recommendation to use DRI in addition to ACEi/ARB or ACEi+ARB combinations and questions the use of MRA for patients with diabetic kidney disease.”

2. **Introduction**

*In the first sentence, "detrimental" could be replaced with "debilitating" or "harmful" to avoid repetition of the same word in the sentence.*

- We exchanged this word with “harmful”.

*In the second sentence, it could be clarified that the increasing incidence of DKD is a global trend, not limited to a specific region or country.*

- We added “globally” to the sentence:

“The incidence of DKD has globally substantially increased over the past three decades.”

*In the third sentence, "considerable advantages" could be elaborated upon to provide more specific details, such as reduction in proteinuria, preservation of kidney function, or decrease in cardiovascular events.*

- We provided a more detailed description of considerable advantages:

“The incidence of DKD has globally substantially increased over the past three decades (2), which lead to the inevitable necessity to develop novel effective drugs to preserve kidney function, decrease the cardiovascular risk or even prevent its onset.”

In the fourth sentence, "mainly dual RAAS blockade" could be revised to "primarily dual RAAS blockade" for greater precision and concision.

- Thank you for the advice. The word “mainly” was replaced by “primarily”.

*In the fifth sentence, it could be helpful to briefly define SGLT2i and nsMRA for readers who may not be familiar with these terms, or to include a footnote or reference to explain them.*

- Noted. We added for each mentioned drug an additional sentence.

SGLT2i block the carrier protein sodium-glucose linked transporter 2, which induces glucosuria and by that initiating a cascade positively affecting the cardiovascular system and kidney. nsMRA (e.g. finerenone, esaxerenone) bind highly selective on mineralocorticoid receptors and thus antagonizes the effect of aldosterone, which is assumed to play a pivotal role in the development of chronic kidney disease.

*In the sixth sentence, "best suitable treatment" could be changed to "optimal treatment strategy" for greater clarity and professionalism.*

- The segment “best suitable treatment” was replaced by “optimal treatment strategy”.

*Additionally, it may be useful to specify the patient population or criteria for inclusion in the analysis, such as stage of DKD or presence of comorbidities.*

- Thank you for the suggestion. We specified our inclusion criteria in the section: methods (page 7, paragraph 2). We did not define the stage of DKD nor presence of comorbidities. But we did exclude patients on dialysis caused by a different disease than DKD.

3. **Method**

It would be helpful to state the date range of the search for articles in Medline and clinicaltrials.gov.

- We added the range from 1995 “till May 2022”.

*The meaning of "nsMRAs" is unclear and should be defined.*

- We did explain the abbreviation nsMRA in the introduction. But we added now which exact drugs we searched for. (finerenone, esaxerenone) (page 7, paragraph 2)

*The exclusion of studies with insufficient information should be clarified (e.g., what was considered "insufficient"?).*

- Thank you for the advice. We added a sentence (page 8, paragraph 2):

We considered as insufficient information, if a study misses for our analysis relevant outcome data, did not state if it only included patient with DKD or did not fully describe the study design (e.g. randomized, controlled).”

*The description of the screening process could be more detailed, including how conflicts were resolved.*

- We included :

“Conflicts over study inclusion and data extraction were resolved by discussion with the third reviewer. “

*It would be helpful to specify the types of missing data and how study investigators were contacted.*

- Missing data: We added more details on that matter. (e.g. authors did not state the background therapy or did not give enough details about study participants.) (page 8, paragraph 3)
- “Contacted via e-mail” was added.

*The description of outcomes could be more succinct and organized.*

- Thank you for your advice. We did make some minor changes to this section. If you have any specific suggestions concerning its structure, we are happy to incorporate them.

*The meaning of "comparison adjusted funnel plots" is unclear and should be defined.*

- We added one more citation for this matter (Evaluating the Quality of Evidence from a Network Meta-Analysis; Georgia Salanti,**,** Cinzia Del Giovane, 2014) and gave a brief explanation about this subject. The sentence says now (page 9, paragraph 2)

: “… and additionally reporting bias through comparison adjusted funnel plots which was applied to compare older treatments with newer treatments.”

“In such a plot, the vertical axis represents the inverted standard error of the effect sizes as in a standard funnel plot. However, the horizontal axis represents an adjusted effect size, presenting the difference between each observed effect size and the mean effect size for the specific comparison being made. The use of such a plot is informative only when the comparisons can confidently be ordered in a meaningful way; for example, if all comparisons are of active treatment versus placebo, or all are of a new versus an old drug. Examination of any asymmetry in the plot can help to infer about the possible presence of an association between study size and study effect. “ Salanti et. al. 2014 (Evaluating the Quality of Evidence from a Network Meta-Analysis)

*It would be helpful to specify the statistical significance level used for Cochran's Q statistics.*

- We added the significance level of 0.05.

*The rationale for the sensitivity analyses should be explained more clearly.*

- We modified the respective paragraph to better explain our rationale. It now reads (page 10, paragraph 2).

“To assess the robustness of our network analysis, we conducted several sensitivity analyses(8) in order to determine how our results are affected by different input variables. Firstly, we included only studies with low and moderate risk of bias (supplementary S8) to examine the influence of different levels of risk of bias on our results. Additionally, we performed a second sensitivity analysis, including only studies in which single RAAS treatment was mandatory in the control group. This excludes studies, in which only our 80% criterion regrading ACEi/ARB background treatment was fulfilled and not all patients were receiving ACEi/ARB treatment. By changing these specific input variables, we investigated how crucial our results are impacted by our decision to include studies without a mandatory ACEi/ARB background therapy. “

*There are some grammar and syntax errors (e.g., "We considered 'background treatment with single ACEi/ARB' as fulfilled," "Furthermore, image extraction software... was used for relevant outcome data presented only in form of figures without numerical values.").*

- Changes were implemented.

“If a minimum of 80% of patients in a study were taking either ACEi or ARB, our criteria “background treatment with single ACEi/ARB” was fulfilled. “ (page 7, paragraph 2)

For relevant outcome data presented only on form of figures without numerical values, we applied an image extraction software (digitizeit 2.5.; I. Bormann, Braunschweig, Germany). (page 8, paragraph 3)

4. **Results**:
*In the sentence "Merely additional SGLT2 inhibitors on top of ACEi/ARB vs. single ACEi/ARB revealed a significant effectiveness in reducing the risk for ESKD," "Merely" should be replaced with "Only" or "Just".*

- Changes were implemented.

*In the sentence "Comparison of the interventions with each other did not show any significant difference (Table 1).", "Table*

- The sentence now:

Comparison of these interventions with each other did not show any significant difference.

*In the sentence "The sensitivity analysis, including only clinical trials stating single ACEi/ARB as active control displayed the same trend as without any pre...", the sentence is incomplete and needs to be finished.*

- Rephrased.

Both sensitivity analysis displayed a similar trend as without any exclusions.

*It will be better to show kappa for the selection and data extraction. Please show the data of kappa of agreement during the systematic searches. How disagreements were solved during the systematic search among two independent reviewers?*

- We calculated kappa inter-rater reliability with R. (page 17, paragraph 3)
- screening level: kappa = 0.88
- abstract level: kappa = 0.89

“Conflicts over study inclusion and data extraction were resolved by discussion with the third author (BS).” (Page 8, paragraph 3)

5. **Discussion**:
*The statement that SGLT2i is the only drug that caused a decrease in mortality and the incidence of ESKD is not correct. The discussion did not mention which drugs were compared in the network meta-analysis, but it is unlikely that SGLT2i was the only drug that showed a positive effect. This statement needs to be revised.*

- We agree, this statement was overreaching. We changed it to (Page 21, paragraph 2)

“In conclusion, SGLT2i emerged in our analysis as the only drug class showing beneficial effects on top of ACEi/ARB treatment regarding mortality and end stage kidney disease and by that reconfirmed its position as treatment option for diabetic kidney disease. nsMRA did not raise any safety concerns and diminished the risk for our renal composite outcome, justifying its application. “

*The discussion states that the positive effect of SGLT2i on DKD has been comprehensively reviewed recently, citing a reference (11). However, the reference provided does not seem to be related to the positive effects of SGLT2i on DKD. This needs to be clarified.*

- We added a different reference. (SGLT2i: beyond the glucose-lowering effect, [Lihua Ni](https://cardiab.biomedcentral.com/articles/10.1186/s12933-020-01071-y#auth-Lihua-Ni) et. al. 2020)

*The discussion claims that dual blockade with ACEi and ARB and the addition of aliskiren have been completely banned due to major safety concerns. This is not entirely correct. While some studies have shown increased risks of adverse events with these combinations, they are not completely banned and may still be used in certain circumstances.*

- We agree. We also use ARB and ACEi in combination or especially ACEi/ARB plus aliskiren in selected cases. We modified the respective sentence (Page 19, paragraph 1):

Unfortunately, the dual blockade with ACEi and ARB as well as the addition of aliskiren raised major safety concerns in large randomized, controlled trials(4)(23) which led to a restricted use of these two combination therapies.

*The discussion suggests that MRAs have been the only treatment alternative for patients with progression of DKD and/or gross proteinuria despite ACEi/ARB and SGLT2i treatment. This is not entirely accurate as there are other treatment options, such as GLP-1 receptor agonists, which have shown promising results in clinical trials.*

- We agree and mention now GLP-1 receptor antagonists. The sentence reads (Page 19, paragraph 2)

“Until recently MRAs besides Glucagon-like peptide-1 receptor agonists were the only treatment alternative for patients with progression of DKD and/or gross proteinuria despite ACEi/ARB and SGLT2i treatment.”

*The discussion states that nsMRAs tend to show a lower incidence of hyperkalemia compared to steroidal MRA. However, the evidence is not conclusive, and further studies are needed to confirm this claim.*

- We agree that this sentence alone might have been misleading. We added a sentence to clarify this concern (page 20, paragraph 1)

“Our results are not confirmative for this suggested correlation and require more studies for validation.”

*The discussion claims that ongoing studies are exploring the combined effect of finerenone and empagliflozin. However, the reference provided for this claim is outdated, and newer studies have been published on this topic.*

- To our knowledge there are no published clinical trials that examine the combination of finerenone and empagliflozin/dapagliflozin. We are happy to add a study we have missed following your advice.

**Journal requirements:**

1. Please ensure that your manuscript meets PLOS ONE's style requirements, including those for file naming. The PLOS ONE style templates can be found at

- Requirements were implemented.

2. Please include a separate caption for each figure in your manuscript.

- Separate caption for each figure were incorporated.

3. Please include your tables as part of your main manuscript and remove the individual files. Please note that supplementary tables (should remain/ be uploaded) as separate "supporting information" files

- Implemented.

4. In your Data Availability statement, you have not specified where the minimal data set underlying the results described in your manuscript can be found. PLOS defines a study's minimal data set as the underlying data used to reach the conclusions drawn in the manuscript and any additional data required to replicate the reported study findings in their entirety. All PLOS journals require that the minimal data set be made fully available. For more information about our data policy, please see <http://journals.plos.org/plosone/s/data-availability>.

- We added a R -code and data sheets for each endpoint in our supplementary.
