# Supplementary material for: Treatment of diabetic kidney disease. A network meta-analysis
Source: PLoS One. 2023 Nov 2;18(11):e0293183. doi: 10.1371/journal.pone.0293183 (PMC10621862; doi:10.1371/journal.pone.0293183)
Supplement: Attachment — Submitted filename: rebuttal letter_2_BS-2.docx [file pone.0293183.s020.docx]

Dear editor,

We thank the reviewers again for their efforts.

We have addressed the additional comment of Reviewer #1.

Reviewer #1:

*The authors have failed to include the FIGARO trial Pitt B et.al. NEJM and more importantly the FIDELITY individual patient analysis that includes over 13000 patients-this is NOT a meta-analysis as it is all patients used from two trials with the same protocol and centers but different inclusion criteria Agarwal R et.al. Eur Heart J 2022. Additionally data from this analysis does show a benefit on all cause mortality.*

In our primary analysis we had separately included the FIGARO and FIDELIO trials (compare supplemental table 1). We did not include the FIDELITY pooled analysis, as this uses the same data. Now, we performed an additional sensitivity analysis treating FIDELITY as one trial and included it into the analysis, while we omitted FIGARO and FIDELIO. This analysis (given as new supplemental figure 15) gives us essentially the same results.

Supplemental figure 12 a OR of mortality with using FIDELITY as a single study

Supplemental figure 12 b OR of ESKD with using FIDELITY as a single study

In addition we mentioned this analysis in the paragraph of the methods section on sensitivity analyses on page 10, paragraph 2

Finerenone was examined in two major clinical trials (FIGARO, FIDELIO). In our primary analysis we included these separately. However, as these trials had an identical design and were published also pooled in a prespecified analysis (FIDELITY), we repeated our analysis by treating the data from FIDELITY as single trial but excluding Figaro DKD and Fidelio DKD.

We changed the paragraph on sensitivity analyses in the results section (page 17, second paragraph):

All three sensitivity analyses displayed a similar trend as without any exclusions (S10, S11, S 12). However, the effect of nsMRA on ESKD was somewhat larger in the analysis treating FIDELITY as single trial resulting in a significant result (OR 0.80, 95% CI 0.64 - 0.99).

In addition, we rephrased a sentence in the discussion on page 20 first paragraph:

Whereas SGLT2i have already emerged as standard treatment of diabetic kidney disease [28] nsMRA are recommended in patients with persisting microalbuminuria despite sufficient ACEi/ARB treatment as additional treatment in the current consensus report of the American Diabetes Association and Kidney Disease: Improving Global Outcomes (KDIGO) (29) and the KDIGO 2022 Clinical Practice Guideline for Diabetes management in Chronic Kidney Disease (30).

*Moreover, not sure why Exacernone is included as there No trial data other than albuminuria in diabetic nephropathy.*

We included esaxerenone as the study mentioned fulfilled our inclusion criteria and contributed to a secondary endpoint of our analysis. In order to show the complete evidence, we would like to leave this in.

*Hence, this is not a complete analysis.*

We do not agree. Our analysis is complete.
