# Supplementary material for: Treatment of diabetic kidney disease. A network meta-analysis
Source: PLoS One. 2023 Nov 2;18(11):e0293183. doi: 10.1371/journal.pone.0293183 (PMC10621862; doi:10.1371/journal.pone.0293183)
Supplement: Attachment — Submitted filename: Rebuttal letter 23.7.23.docx [file pone.0293183.s021.docx]

Dear editor,

We thank the reviewers again for their efforts.

We have addressed the additional comments of Reviewer #2 and believe the manuscript is now appropriate for publication in PLOS ONE.

The **introduction** contains relevant background information on the topic. However, it's slightly unclear due to the usage of technical terms without providing a clear definition or explanation.

1. Improvement: Make sure to clearly define all the terms and abbreviations you are using. Also, provide a clear research question or objective to give the reader an idea of what you aim to achieve with your study.

- Thank you for pointing this out. We added some explanations to several scientific terms (e.g. RAAS).

RAAS is a hormonal system that regulates the fluid and electrolyte balance and the systemic vascular system in the human body through various pathways, that affect the kidney. (Page 5, paragraph 2)

…. aldosterone, which is a steroid hormone and assumed to play a pivotal role in the development of chronic kidney disease. (Page 6, paragraph 3)

- In addition, we rephrased our objective to emphasize our goals and further elaborated our research question. (Page 6, paragraph 4)

Our objective is to identify the optimal treatment strategy in addition to RAAS blockade by ACEi/ARB for patients with DKD. We mainly focused on the following questions:

- Which drug most effectively reduces the risk of our primary endpoint?
- Which drug has the lowest risk of causing the side effects we have specified?

**Method**
There seems to be missing detailed explanation about the methods used to conduct the study. The methods section should provide enough details for someone else to reproduce your study.

2. Improvement: If applicable, describe the design of the study, the population studied, the treatments or interventions, and the type of statistical analysis. For example, how did you perform your network meta-analyses? What were your inclusion and exclusion criteria?

- Thanks for the advice. We explained the technique of network meta-analysis in more detail.

Network meta-analysis is a statistical method for directly or indirectly comparing multiple interventions. It allows us to calculate the comparative effectiveness of interventions that have not been studied in head-to-head trials or have not been adequately studied. For each intervention, we applied the random effect model to generate the study effect sizes. (Page 9)

Data analysis was performed with the statistic program R version 4.0.4 using the “netmeta” package. (Page 9)

- We also added some references in the manuscript to our R code and the corresponding datasheet that we used to conduct the network meta-analysis.

The specific code and data sheet for our network meta-analysis can be found in the supporting information (S14, S15). (Page 9)

- We also defined eGFR and GRADE to make the manuscript more accessible to people outside the field.

eGFR (estimated Glomerular Filtration Rate) is a measure for the estimated kidney excretory function. (Page 9)

Moreover, we rated the quality of evidence of each pairwise comparison according to GRADE (Grading of Recommendations Assessment, Development and Evaluation) (Page 11)

**Results**
The results are shared but might be unclear for people outside the field. For instance, the term 'renal composite outcome' is used but not defined.

3. Improvement: Be clear and concise about the results. Use common language to explain technical terms where possible. Make sure to provide context and definitions for terms like 'renal composite outcome'.

- We reevaluated our results section and implemented several changes:

Figure 2 shows the network structure for the primary outcome overall mortality, highlighting the larger number of clinical trials for SGLT2i, nsMRAs and ACEi + ARB. (Page 12)

eGFR (estimated Glomerular Filtration Rate) is a measure for the estimated kidney excretory function. (Page 9)

- The term “renal composite outcome” was defined in our results.

The renal composite outcome consisting of sustained eGFR of less than 15 mL/ min/1.73 m2, sustained eGFR decline of 40% from baseline or kidney death was available for SGLT2i and nsMRAs, both reported by two trials each.

**Discussion**
The discussion has relevant insights but jumps between different studies and results quite abruptly.

4. Improvement: Provide more coherent and fluid connections between different points. Compare your results with previous studies in a structured way, emphasizing the novel contributions of your work. Also, the implications of your findings should be spelled out more clearly for the readers.

- Thank you for the advice. We have made several changes:

However, steroidal MRAs, i.e. spironolactone and eplerenone, were disproportionally underrepresented for sufficient data on hard endpoints in our analysis.

A further evolution of MRA, nsMRAs are the most recently developed pharmacological agents acting on the RAAS system.

Unlike ACEi+ARB combination or MRAs, nsMRA did not cause higher rates of acute kidney injury (compared to single ACEi/ARB).

5. In all sections, remember to provide brief explanations of cited studies, such as the key findings or methodologies of the referenced works. Moreover, do edit for grammar and style to ensure the clarity and readability of the text.

- The whole manuscript was proofread by a native speaker again.

Risk of bias was assessed using the RoB 2 tool (The Cochrane Collaboration’s tool for assessing risk of bias) and additionally reporting bias through comparison adjusted funnel plots which was applied to compare older treatments with newer treatments.

In our analysis, they were associated with a lower incidence of the renal composite outcome (compared to single ACEi/ARB).
