# Supplementary material for: Treatment of diabetic kidney disease. A network meta-analysis
Source: PLoS One. 2023 Nov 2;18(11):e0293183. doi: 10.1371/journal.pone.0293183 (PMC10621862; doi:10.1371/journal.pone.0293183)
Supplement: Attachment — Submitted filename: rebuttal letter.docx [file pone.0293183.s022.docx]

Dear editor,

We thank the reviewers again for their efforts.

We have addressed the additional comments of Reviewer #2 and believe the manuscript is now appropriate for publication in PLOS ONE.

**Reviewer #2:** Areas for Enhancement:
1. Figure 1: Adopt a formal PRISMA flow diagram for clarity.

- Thank you for pointing this out. We adopted the PRISMA flow diagram to the formal requirements.

2. Introduction: Ensure technical terms and abbreviations are well-defined to enhance clarity for all readers.

- We did make several adjustments.
  - “Diabetes mellitus (DM) ranks among the top ten most devastating diseases worldwide, ….” (Page 5/ Paragraph 1)
  - Single RAAS blockade was replaced by “Drugs based on a single RAAS Blockade in particular angiotensin-converting enzyme inhibitor (ACEi) or an angiotensin receptor blocker (ARB), were the only pharmacological approach to show considerable advantages” (Page 5/ Paragraph 2)
  - Rephrased a sentence: “The evidence for the benefit of an additional RAAS blockade agent, either by combining ACEi+ ARB or one of them with e.g. mineralocorticoid receptor antagonists (MRA) or a direct renin inhibitor (DRI), remained limited.“ (Page 5/ Paragraph 2)
  - Acute kidney injury was defined. “(abrupt changes in kidney function, including serum creatinine changes and urine output within 48 hours or 7 days) KDIGO” (Page 5/ Paragraph 2)

3. Objective: Clearly articulate the study's purpose to guide readers.

- We rephrased the study´s purpose (Page 6):
  - Our objective is to identify the optimal treatment strategy in addition to ACEi or ARB for patients with DKD. We mainly focused on the following questions:
    - Which drug most effectively reduces the risk of overall mortality, the development of end stage kidney disease, acute kidney injury, hyperkalemia and hypotension?
    - Which medication carries the least risk of inducing the specific side effects we have outlined?

4. Methods: Offer a more detailed account of the techniques employed to facilitate study replication.

- We added the following information:

The following characteristics were extracted study name, registration number, background therapy, type of study, inclusion criteria, exclusion criteria, study duration, study drug, dosage, mean age, percentage of male patients, outcome data and history of hypertension. (Page 8/ Paragraph 2)

- We did incorporate several new explanations to specific R-packages and its functions.
  - We computed the network plot with the “netgraph” function from the “netmeta” package, the comparison-adjusted funnel plots with the function “funnel” and the splitting of direct and indirect evidence with the “netsplit” function. (page 10 paragraph 2)
  - The I^2^ value were computed with the “netmeta” function. (page 10/ paragraph 3)
- Additional information has been added.
  - “Risk of bias was assessed by two authors using the RoB 2 tool.” (page 10/paragraph 1)
- The search strategy and the corresponding results may be found in the supporting information document under S4.

5. Network Meta-analysis: Elaborate on the statistical methods and tools utilized for a comprehensive understanding.

- We further elaborated on the terms: design inconsistency, heterogeneity, net heat plot and loop inconsistency.
- Net heat plot
  - The gray squares represent the degree of importance of one treatment comparison for the estimation of another treatment comparison and the color in the background illustrates the degree of inconsistency. (page 10, paragraph 3)
- design inconsistency & loop inconsistency
  - Design inconsistency stands for discrepancies in effect estimates between studies involving different composition of interventions. Loop inconsistency evaluates if direct and indirect evidence correspond with each other (9). (page 10, paragraph 3)
- Heterogeneity
  - Heterogeneity refers to the variability or differences in effect estimates across studies within a network (9). (page 10, paragraph 2)

6. R Code References: Incorporate references to the R code and associated datasheets to bolster transparency.

- We did incorporate several new explanations to specific R-packages and its functions.
  - We computed the network plot with the “netgraph” function from the “netmeta” package, the comparison-adjusted funnel plots with the function “funnel” and the splitting of direct and indirect evidence with the “netsplit” function. (page 10 paragraph 2)
  - The I^2^ value were computed with the “netmeta” function. (page 10/ paragraph 3)
- The specific R-code and data sheet for our network meta-analysis can be found in the supporting information (S14, S15).

7. eGFR Definition: Clearly define eGFR (estimated Glomerular Filtration Rate) for broader audience comprehension.

- eGFR (Estimated Glomerular Filtration Rate) is a clinical measurement used in nephrology to estimate the rate at which the glomeruli are filtering waste products and excess substances from the blood per unit of time. (Page 9)

8. GRADE Rating: Utilize the GRADE system to assess the quality of evidence for each comparison.

- We have included the data related to the quality of evidence (GRADE system) for hypotension and the composite outcome in the supporting information S16. The GRADE assessment for the remaining interventions can be found in tables T1 and T2, making the GRADE assessment accessible for all comparative analysis.

Potential Concerns:
1. Terminology: Ambiguous or undefined technical terms can confuse readers.

- We reevaluated the manuscript for ambiguous or undefined terms. (e.g. We switched in the introduction “single RAAS blockade” for “drugs based on a single RAAS blockade” to improve the understanding, added a definition for acute kidney injury)
- If there are other terms that are not defined, feel free to point them out.

2. Methods Detailing: An insufficiently detailed methods section can compromise study reproducibility.

- We elaborated further on details concerning the methods. (e.g. additional explanation of R -packages and which are used, several other details were added)

3. Analysis Transparency: Omitting specifics about analytical tools and methods can cast doubts on the study's credibility.

- We have implemented concerns/comments from the “area for improvement” and added more details to the methods section. Our whole R-code and the corresponding data may be found in the supporting information file under S4.

**Additional Editor Comments:**

Edit for grammar and style to ensure the clarity and readability of the text

- Noted. We did implement several changes related to grammar and style.

Abstract Please provide info re; methodology

- Risk of bias, GRADE approach were added.

Control groups: where is this patient on ACE//ARB only? Please clarify. What do you mean by placebo?

- In most clinical trials we assessed, the intervention of interest (e.g., SGLT2i) was typically compared to either an angiotensin-converting enzyme inhibitor or an angiotensin receptor blocker, and in some cases, a placebo might have been included as well. We referred to this combination as an active control. No clinical trials solely employed a placebo. However, the most common combinations involved ACEi/ARB with either a placebo or ACEi/ARB alone. Our control group consists of mostly an active control as ACEi or ARB drug combination.
- We did not specify placebo any further.

Hypothesis could be restated to better reflex the study

- The hypothesis underwent a revision (Page 5).

Citation for statistic program needed.

- Citation was added. (Page 9)

Please remove figure legends from results sections, hard to read

- Implemented. The corresponding figure legends may be found at the end of the document. (Page 26)
